# Modelling the sustainability of a primary school digital education curricular reform and professional development program

**DOI:** 10.1007/s10639-023-11653-4

**Published:** 2023-06-15

**Authors:** Laila El-Hamamsy, Emilie-Charlotte Monnier, Sunny Avry, Morgane Chevalier, Barbara Bruno, Jessica Dehler Zufferey, Francesco Mondada

**Affiliations:** 1 MOBOTS Group, Ecole Polytechnique Fédérale de Lausanne, Lausanne, Switzerland; 2 LEARN – Center for Learning Sciences, Ecole Polytechnique Fédérale de Lausanne, Lausanne, Switzerland; 3 University of Teacher Education (Haute Ecole Pédagogique) Vaud, Lausanne, Switzerland; 4 Computer Human Interaction in Learning and Instruction (CHILI) Laboratory, Ecole Polytechnique Fédérale de Lausanne, Lausanne, Switzerland

**Keywords:** Educational change, Sustainability, Professional development, Digital education, Curricular reform, Primary school

## Abstract

Sustaining changes in teachers’ practices is a challenge that determines the success of curricular reforms, from which Digital Education (DE) is not exempt. As the literature on sustainability is considered “scarce” and “scattered”, long-term studies modelling the factors impacting teachers’ sustained uptake of DE pedagogical content are lacking. Thus, we investigate whether and how 287 in-service teachers sustained a primary school DE curricular reform over a year after they completed their two-year DE professional development program. We model the sustainability of the reform through Structural Equation Modelling, and identify critical sustainability-factors. The validated Sustainable Adoption of Digital Education (SADE) model confirms that sustainability in the fourth year of the reform depends on perceived usefulness of teaching the new content, ease of implementation, and access to sufficient support in schools. Such factors should thus be evaluated, accounted for in the implementation phase of the reform, and sustained over time. The findings confirm that the DE curricular reform model contributes to positive self-efficacy to teach DE, provides sufficient in-school support, and promotes increasing adoption over time. However, as teachers’ practices have not yet stabilised, and teachers may still adopt more to cover the breadth of DE-concepts, it is important to remain attentive to remaining sustainability barriers: lack of time, effort required to teach DE with teachers preferring to delegate, and lack of student-learning evidence, the latter being a significant challenge to address in the literature. These barriers must therefore be jointly addressed by researchers and practitioners in the field in order to promote the sustainability of the reform.

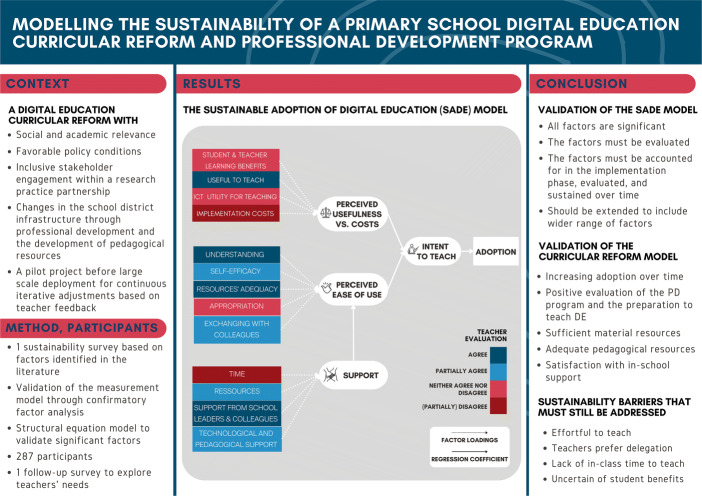

## Introduction

### The challenge implementing and sustaining large-scale curricular reforms

The objective of curricular reforms is to bring about scalable and sustainable changes in teachers’ practices that contribute to improving education (Jamaludin & Hung, 2016, p.361). Unfortunately, large-scale curricular reforms do not automatically result in long-term changes in teachers’ practices (Tikkanen et al., 2020), even when initial implementations are successful (Shirrell & Spillane, 2020). In fact, many curricular reforms have failed to reach the classroom and influence instruction (Coburn, 2003, p.2). This is not surprising since “large-scale school reforms are highly complex processes, and their success is regulated by multiple factors (Shirrell & Spillane, 2020) at different levels of the education system, ranging from the national level to the classroom level” (Tikkanen et al., 2020, p.546). Unfortunately, sustainable change is considered to be “one of the biggest challenges in education” (Hubers, 2020) as teachers often make “superficial” (Hubers, 2020), and “short-lived” changes to their practice, “reverting back to their ‘old ways’ after funding and support are withdrawn” (Lee & Louis, 2019, p.85), hence the complexity of sustaining a reform.

As sustainability is a prerequisite for scaling educational endeavours (Coburn, 2003; Howard et al., 2021), and sustaining changes in teachers’ practices is a considerable challenge, it is essential to understand how to improve the sustainability of curricular reforms. Unfortunately, the sustainability literature is “scarce” and “scattered” (Hubers, 2020), with “little [being] known about the dynamics of sustaining change in school reform and how the process of change unfold[s]” (Li, 2017, p. 279), despite decades of research (Coburn, 2003; Li, 2017). That is why researchers are calling for “more knowledge about how and why changes were (not) sustained over time” (Hubers, 2020, p.10) through longitudinal studies and investigations targeting later phases of reforms that are currently under-researched (Coburn et al., 2012; Li, 2017; Howard et al., 2021). Finally, since sustainability factors are highly context dependent (Gersten et al., 2000; Harris & Jones, 2018; Kampylis et al., 2013), studies must be conducted in the context of the reform and the practice intended to be sustained.

### The case of digital education for primary school

Digital Education (DE), which includes Computer Science (CS, and Computational Thinking - CT), Information and Communication Technology (ICT, including Digital Literacy), and Digital Citizenship (El-Hamamsy et al., 2021b), has been the subject of numerous curricular reforms worldwide in recent years, and is no exception to implementation and sustainability struggles. One could even stipulate that these issues are more prominent for DE, and in particular in primary school where teachers often teach all subjects, with specific time for DE not always being allocated. Studies have found that primary school teachers already struggle with science-related subjects (Drits-Esser et al., 2017, p. 377; Hubers et al., 2020). Digital education, as a new field, therefore requires that teachers first learn the fundamental concepts and how to use the underlying tools (first stage of instrumentation, Trouche, 2005) before being able to teach them (second stage of instrumentation, Trouche, 2005), an approach that was already successful for in-service teacher education (Repenning et al., 2019). Therefore, it is surprising to find that many DE-related studies have referred to the “barriers [that need to be] overcome [to] [implement] a high-quality, valued and sustainable Computer Science curriculum, [and ensure] that there is the confidence and capability in the teaching profession to deliver it effectively (Passey, 2017)” (Moller & Crick, 2018, p. 416). Similar statements have been made regarding the difficulties teachers face teaching ICT (Heitink et al., 2016; Passey, 2017; Niederhauser et al., 2018), which is likely due to the under-preparation of pre-service teachers (Farjon et al., 2019, p.82), and insufficient professional development (PD) provided to in-service teachers (The Royal Society, 2017; 2012). Add to this that teachers are generally reluctant to adopt instructional or curricular innovations, specifically those related to technology, as technology is perceived to be constantly changing (Ertmer & Ottenbreit-Leftwich, 2010). At a time when DE is part of curricula worldwide (European Education and Culture Executive Agency and Eurydice, 2019; Bocconi et al.,^2022^), with increased access to digital tools in schools, many consider that “little has changed in the classrooms” (Redmond et al., 2021, p. 2895), with the use of technology remaining superficial (Niederhauser et al., 2018). The difficulties are further exacerbated by the lack of consensus on the effectiveness of DE related reforms (Toh, 2016, p. 146), raising numerous questions about the sustainability, impact, and costs of these initiatives. Unfortunately, “the complexity of interacting factors impacting scalability^1^ and sustainability raises numerous challenges relating to technology integration initiatives and innovation” (Niederhauser et al., 2018). Therefore, the prognosis mirrors that of sustainability more generally: “considerably more research needs to be done to understand how successful technological innovations and change processes are sustained and scaled to new learning contexts” (Howard et al., 2021, p. 2309).

To the best of our knowledge, most studies on DE related reforms and PD programs either do not assess the change in behaviour (here, adoption of curricular content), or do not have insight into what is done over time and after PD programs have ended (El-Hamamsy et al., 2022a; Howard et al., 2021), let alone evaluate the depth of the change in teacher practices (Coburn, 2003). Howard et al., (2021) have therefore called for a change in sustainability research because “although these studies provide valuable new insights [into] school reform as a journey, we still know little about how this journey unfolds, especially after the withdrawal of external support and during later phases of reform (Coburn, 2003; Coburn et al., 2012)” (Li, 2017). Although the literature evokes numerous factors that impact sustainability (see Section 3), to the best of our knowledge, we lack insight into how and to what extent sustainability factors interact to impact the long-term sustainability of a DE curricular reform. Considering the extent of the change brought on by DE curricula, particularly in primary school where teachers must teach all disciplines, it becomes paramount to investigate these factors.

### Problem statement and research questions

Building on the challenges of investigating sustainability (discussed in Section 1.1) and particularly in the case of DE curricular reforms (discussed in Section 1.2), we focus on the case of a DE curricular reform. The objective is to investigate ‘if, under what circumstances, and how [the reform] has been successfully sustained” (Howard et al., 2021), where sustainability refers to teachers continuing to satisfactorily implement the reform over time, without requiring overt efforts on their part (see Section 3.1). By analysing data acquired from 287 primary school teachers (grades 1-4) in the fourth year of the DE curricular reform, which is more than a year after having finished their mandatory two-year DE Professional Development (PD) program, we therefore look to contribute to the literature on sustainability of DE curricular reforms by addressing the following research questions:

**(RQ1)**: What factors significantly influence the sustainability of the DE curricular reform in the fourth year, i.e., more than a year after the end of the two-year DE-PD program?
**(RQ2)**: Has sustainability of the DE curricular reform been reached in the fourth year, i.e., over one year after the end of the two-year DE-PD program?

The analysis is timely as i) it typically takes two years for teachers to master a new practice (Gersten et al., 2000), ii) time is needed to establish communities of practice, see other teachers implement the content, and perceive the benefits (Klingner et al., 2001), and iii) sustainability should be evaluated after the end of PD programs (Coburn, 2003).

The study thus provides two main contributions. The first is a statistical model of the factors influencing the sustainability of the implementation of the novel DE curriculum by primary school teachers, which we establish through Structural Equation Modelling. The second is an evaluation of the sustainability of a DE curricular reform and DE-PD program that considered sustainability from the start of the endeavour (see Section 4.1), thus providing insight into the effectiveness of the curricular reform model and the barriers that may still influence sustainability at this stage of the reform.

## Related work

Given the sparsity of the sustainability literature, we report on studies investigating sustainability both outside and within DE contexts in the following sections.

### A dearth of inferential and long-term sustainability studies in the context of curricular reforms

Numerous studies on changes in teacher practices after curricular reforms or PD programs are conducted in the early stages of the endeavours (Sullanmaa et al., 2019; Liou et al., 2019). Few studies evaluate sustained changes in teacher practices over multiple years (Vaughn et al., 1998; Sindelar et al., 2006; Drits-Esser et al., 2017; Wolthuis et al., 2020). Those who did were often qualitative (Ramberg, 2014, p.3) or conducted with small samples (Drits-Esser et al., 2017). Unfortunately, while shedding light on the factors that contribute to the success (Vaughn et al., 1998) or failure (Sindelar et al., 2006) of the endeavours, they do not provide insight into how the influencing factors interact. Additionally, only one study investigated a district-level curricular reform, while others were school-driven. Indeed, the latter are more likely to be supported by school leaders, have a school culture that promotes the reform (Tikkanen et al., 2020), and therefore succeed compared to wide-spread top-down reforms (Sindelar et al., 2006; Eickelmann, 2013; Lee and Louis, 2019). To the best of our knowledge, only one quantitative and inferential study investigated the factors influencing the sustainability of a reform several years later. Using Structural Equation Modelling (SEM) on nation-wide data from 738 teachers 6 years after the curriculum changed, Ramberg (2014) found that the reform moderately influenced teachers’ practices, with school-based conditions and leadership having an impact on changes teachers reported in their practice. Therefore, it would appear that “previous empirical survey studies concerning large-scale school reforms are scarce” (Tikkanen et al., 2020, p.552) with “prior research on school reforms hav[ing] mostly been single or multiple case studies (see Fullan 2016) employing qualitative methodology” (Tikkanen et al., 2020, p.552). Given the novelty of DE in schools, the challenges involved, and that DE reforms are often national or regional, DE offers an interesting context to study sustainability.

### A lack of models for the Sustainability of digital education curricular reforms

Several studies quantitatively investigated whether the introduction of DE into teachers’ practices was sustainable. However, they did not model the relationship between the factors considered and teachers’ sustained implementation of the curriculum. For example, Redmond et al. (2021) evaluated teachers’ implementation of a Digital Technologies curriculum and identified the following barriers through descriptive analyses: time, overcrowded curriculum, and limited access to professional learning. Similarly, 5 years after a research program explored the use of ICT in 6 schools, Eickelmann (2013) investigated the factors that support and hinder its sustainable implementation. The authors triangulated qualitative and quantitative data from multiple stakeholders (principals, IT coordinators, 180 teachers, students) and derived conclusions from commonalities between schools that did not implement ICT sustainably. Their results highlighted the role of school-level factors (namely, support from school leaders and ICT support structures) on the sustained use of ICT in lectures. Finally, Agyei (2021) followed up on a digital-hubs project in sub-Saharan Africa 6 months after an 18-month ICT-PD program with 4945 teachers from 6 countries through surveys and diaries. They found that teachers were satisfied with the PD but lacked “essential conditions to support transfer of the training’s ideas to the school-level” (Agyei, 2021), thus providing recommendations for effective ICT-PD. Therefore, to the best of our knowledge, no study has modelled the relationship between teachers’ long-term uptake of DE and the influencing factors within a mandatory curricular reform.

## A model of Sustainable Adoption of Digital Education (SADE)

### An operational definition of sustainability for digital education curricular reforms

Hubers (2020, p.1) defined sustainable changes in education as “1) substantial changes made that affect the core of educators’ everyday practice; 2) a longitudinal process that begins when educators contemplate making changes and ends when satisfactory achievement on the other characteristics is reached and overt learning efforts are stopped; 3) a process of individual and organisational learning as well as changes in behaviours; resulting in 4) significant positive effects on student outcomes”. In the present study, as the objective is to assess whether teachers are teaching the new DE pedagogical content after having followed a DE-PD to acquire the required Technological Pedagogical Content Knowledge (see Section 4.1), we operationalise Hubers (2020)’s definition for the DE reform context and consider that sustainability refers to:

1. The introduction of the novel DE curriculum into teachers’ practice, implying teaching (i.e., adopting) pedagogical content which is used to teach core DE concepts.
2. The adoption of DE pedagogical content which should start with the DE-PD program, with sustainability being reached when teachers satisfactorily integrate the content into their practice over the long term, without requiring significant efforts on their part (in comparison to other existing content). This change should occur and persist in multiple schools to demonstrate the sustainability of the endeavour (Pieters et al., 2019).
3. The collaboration between teachers and other stakeholders at the school-level (e.g., school leaders and instructional coaches) to support the implementation and sustainability of the change.
4. The endeavour resulting in significant positive effects on student outcomes.

### Indicators of sustainability of changes in teacher practices

To model the relationship between the factors that influence the sustainability of the DE curricular reform, we draw on the factors identified as having an impact on sustainable change in teacher practices from the literature (see Table 2 in Section 4.2). These are put in relation with recommendations regarding the assessment of professional development programs and, in particular, Guskey (2000)’s model which is specific to the context of education. As the target of the present study is to assess the long-term sustainability of introducing DE pedagogical content into teachers’ practices, we consider the following indicators from the teachers’ perspective which draw from Avry et al. (2022)’s operationalisation of Guskey (2000)’s framework. As no model of teacher pedagogical content adoption has to this day been validated at scale (El-Hamamsy et al., 2022a), Avry et al. (2022)’s model operationalises Guskey (2000)’s framework based on two types of models: acceptance of innovation models and the Technological Pedagogical Content Knowledge framework. The models of acceptance of innovation and information systems considered are namely the Technology Acceptance Model (TAM, Davis, 1985; 1989; King & He, 2006) and the Unified Theory of Acceptance and Use of Technology, UTAUT) model. These models, while not specific to education, predict a user’s adoption of technology to their behavioural intention. These models find that behavioral intention in turn is predicted by other factors such as perceived ease of use and perceived usefulness in the case of TAMs; or performance expectancy, effort expectancy, social influence, and facilitating conditions in the case of the UTAUT models. These factors can themselves be further mediated by contextual factors, prior factors and factors suggested by other theories (King and He, 2006). Mishra & Koehler (2006)’s Technological Pedagogical and Content Knowledge framework on the other hand is intended for “curriculum development work in the area of teacher education and teacher professional development around technology”. This framework accounts for the fact that to be able to teach digital education, you need to have acquired specific types of knowledge that are at the intersection between knowledge of technology, pedagogy, and the content itself. As stated by Avry et al. (2022), teachers must therefore acquire “technology knowledge (knowledge about technologies), technological content knowledge (knowledge about how technology and content are related), technological pedagogical knowledge (knowledge about how technology can be used to promote better teaching) and technological pedagogical content knowledge (knowledge about how technology can be used to promote better teaching given the subject matter)”. Drawing from these models, we therefore consider the following indicators for the current study which we present according to the five levels of Guskey (2000)’s framework.

Guskey (2000)’s first level focuses on teachers’ immediate reactions to the PD. While our prior study indicated that the PD-participants were satisfied with the PD format and content (El-Hamamsy et al., 2021b), the teachers’ perception of the training sessions could not be included in the model, as teachers’ responses could not be linked back to previous data collections.

Guskey (2000)’s second level targets teacher-learning as teachers’ mastery of the underlying DE concepts is an important prerequisite to being able to teach them (Trouche, 2005; Mishra and Koehler, 2006; Zehetmeier, 2009; Repenning et al., 2019). As the authorisation to directly assess teachers’ mastery of the concepts was not provided, we considered the following indicators from the teachers’ perspective:

- perceived understanding of the content seen in the training sessions,
- extent to which the pedagogical content was adequate and compatible with their prior practices (Niederhauser et al., 2018, i.e., perceived content validity, Holton et al., 2000), as teachers are more likely to adopt practices that are close to their existing routines (Vaughn et al., 1998),
- self-efficacy (Bandura, 1977) to teach the pedagogical content seen in the PD, an indicator of the adoption of pedagogical content (King & He, 2006; El-Hamamsy et al., 2022a).

Guskey (2000)’s third level focuses on organisational support, that is to say whether the external facilitating conditions support the change in the teachers’ practice which are a “require[ment] for professional development efforts to be successful (Johnson, 2006)”. Indeed, a widespread curricular reform is multilevel and requires considering not only the teacher- and classroom-levels, but also the school- and district-levels which must work together to support the change in teacher practices (Kampylis et al., 2013; Hubers, 2020; Tan and Hung, 2020). In fact, only by affecting these different scales and promoting collective capability can one sustain and scale such changes (Tan & Hung, 2020). Thus, we consider multiple indicators. At the curricular and PD levels, it is essential to provide access to sufficient:

- pedagogical and material resources (Coburn, 2003; Niederhauser et al., 2018; Redmond et al., 2021)
- time (Vaughn et al., 1998; Penuel et al., 2007; Karsenti & Bugmann, 2018; Redmond et al., 2021)

Without access to these resources, even the most motivated teachers will have difficulty integrating the new content into their practices. However, support must also be provided within the schools from:

- School leaders, as they have been found to significantly impact the implementation of reforms in their schools (Toh, 2016; Li, 2017; Niederhauser et al., 2018; Gu et al., 2019; Wu et al., 2019; Howard et al., 2021), either through the school culture (Sindelar et al., 2006; Wu et al., 2019) or by inciting collaborations between teachers (Ramberg, 2014).
- Instructional coaches to provide instructional support and help teachers engage in a supportive professional community (Coburn, 2003; Pieters et al., 2019; Shirrell & Spillane, 2020; Caneva et al., 2023).
- The teacher community (Li, 2017), as teachers can support one another in a community of practice (Coburn et al., 2012; Kampylis et al., 2013; Eickelmann, 2013) either pedagogically or from a technical perspective (Gu et al., 2019), to promote the spread of novel practices in schools (Klingner et al., 2001; Penuel et al., 2007).

Guskey (2000)’s fourth level is related to the participants’ use of new knowledge and skills, which in the present case involves teaching (which we refer to as adopting) the pedagogical content prescribed by the new curriculum. The metric considered thus includes whether the teachers adopted each of the activities they were introduced to during the PD program (i.e., adoption quantity, El-Hamamsy et al., 2021a; El-Hamamsy et al.,2022a).

Finally, Guskey (2000)’s fifth level pertains to student learning outcomes. “To be successful, innovations need to have a clear purpose, the new practices/ expected change and the use of digital technologies need to be valued by practitioners and specifically relate to learning” (Howard et al., 2021). Numerous researchers have indeed stated the importance of providing teachers with evidence of the benefits of introducing the change into their practice (Klingner et al., 2001; Tikkanen et al., 2020; Vaughn et al., 1998; Jamaludin & Hung, 2016; Li, 2017). Although we did not directly assess student learning, we used teachers’ perception of the benefits of teaching DE pedagogical content as *a proxy*. The selected indicators selected targeted the i) perceived utility (King & He, 2006; Venkatesh et al., 2003) of teaching DE and teaching with Digital Tools, and ii) teachers’ perception of costs associated with teaching the discipline (Wigfield & Cambria, 2010).

### Proposing a model of Sustainability of the Adoption of Digital Education (SADE)

**Fig. 1:**
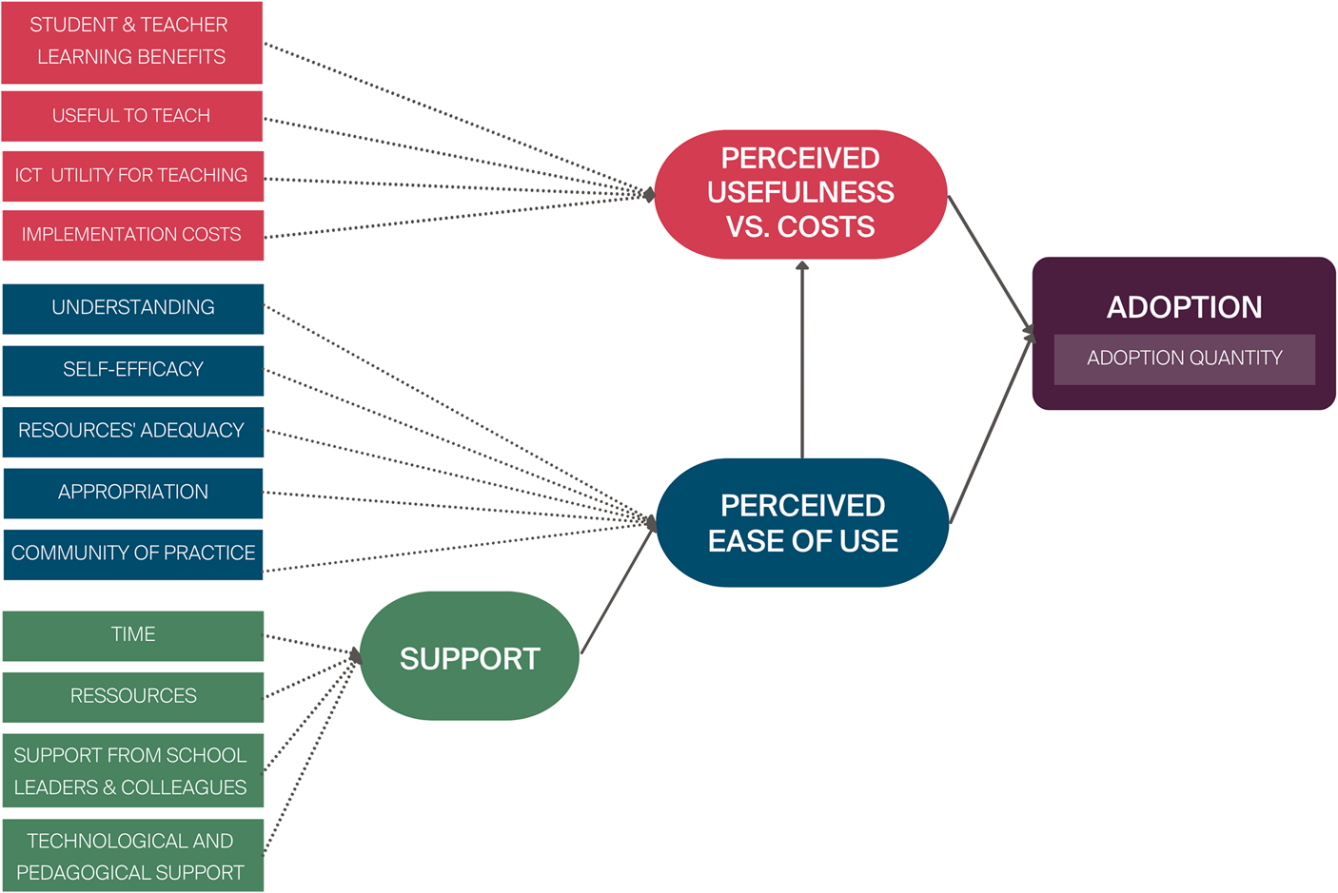
A Sustainable Adoption of Digital Education (SADE) model with corresponding indicators. Latent factors are indicated by the rounded boxes and measured variables by rectangles

Drawing inspiration from Technology Acceptance Models (King & He, 2006), from the literature on technology innovation, and the Teachers’ Adoption of Computer Science (TACS) model (El-Hamamsy et al., 2022a) which adapted TAM models to the context of the adoption of specific pedagogical content, we propose the following model of Sustainable Adoption of Digital Education (SADE) to evaluate the sustainability of a DE curricular reform (see Fig. 1). This model groups Guskey (2000)’s indicators under three factors: perceived ease of use, perceived usefulness, and support. These factors appear in either or both models and are considered predictors of whether or not a user will adopt a new innovation, in addition to being aligned with the sustainability literature.

The *perceived ease of use* factor (blue elements in Fig. 1) comprises of the teachers’ understanding of the content, the adequacy of the pedagogical content with respect to their teaching, their appropriation of the content, their self-efficacy, and whether or not they exchange ideas with their colleagues. The factor *perceived usefulness* factor (red elements in Fig. 1) groups the indicators related to the utility and costs of integrating the discipline into their practices. The *support* factor (i.e., external factors in the TACS model, green elements in Fig. 1) consists of access to sufficient material resources, time, technological & pedagogical support, and support from colleagues & school leaders. The last factor is the actual use of resources (purple elements in Fig. 1) that we refer to as *adoption*, which comprises of the number of different pedagogical activities that the teachers taught over the course of a year without any contact with the DE-PD program providers (i.e., without any official training sessions).

## Methodology

### Context: A digital education curricular reform that put sustainability at the forefront

**Fig. 2:**
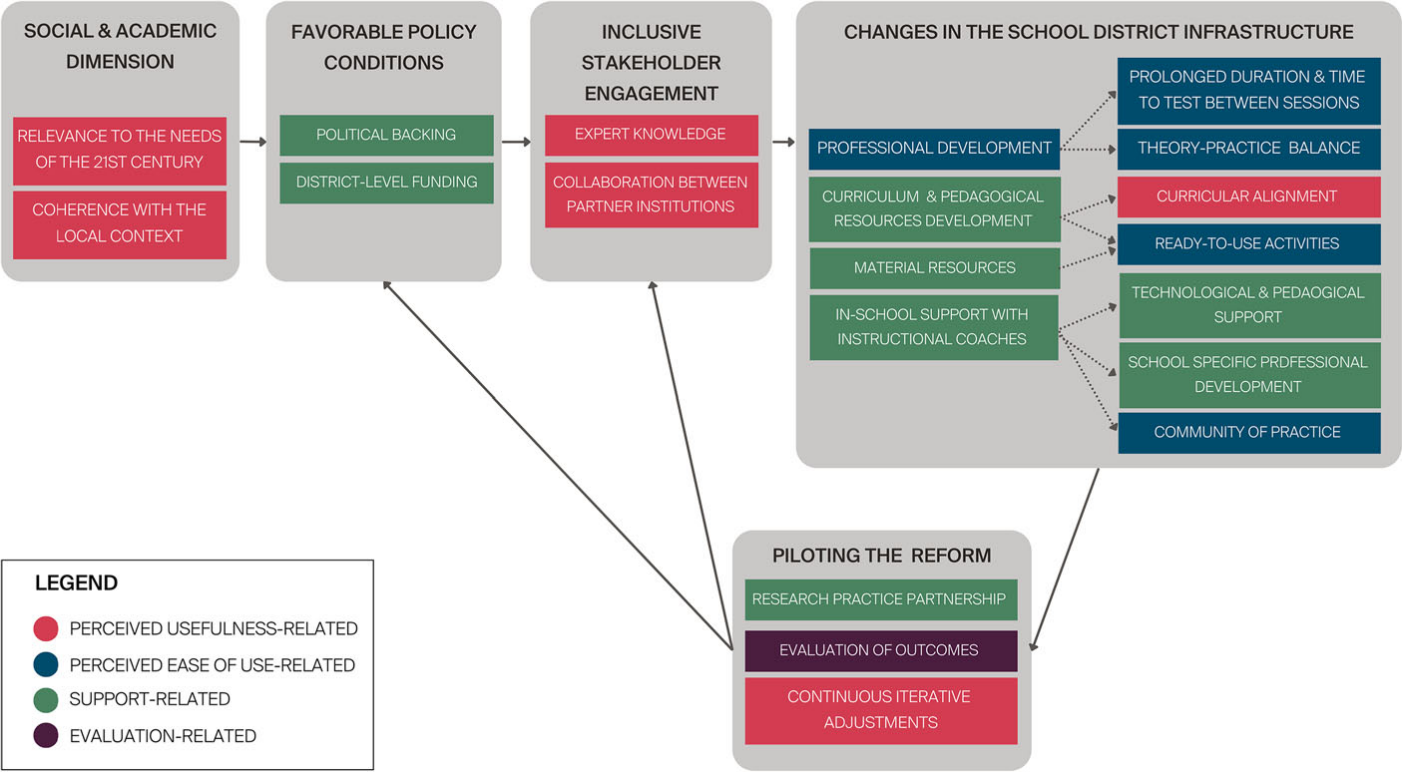
Curricular reform model that looked to promote sustainability and scalability of the endeavour (based on El-Hamamsy et al., 2021b)

The study is carried out within the EduNum project, a mandatory DE curricular reform in the the Canton of Vaud in Switzerland (El-Hamamsy et al., 2021b). This project looks to introduce DE to all K-12 students in the region, i.e., approximately 12’000 teachers and 130’000 students in 93 schools. As such, the project considered sustainability (and scalability) as key issues from the beginning of the curriculum and the teacher-PD design process (see Fig. 2) and accounted for facilitators and barriers to sustaining changes in teacher practices, and teacher PD-best practices. The supporting references are provided below.

Taking into account what are considered to be prerequisites for the sustainability of such endeavours (Fullan, 2001; Toh, 2016; Roesken-Winter et al., 2015; Moller & Crick, 2018), the reform is relevant to the needs of the 21st century, has political support, is fully funded by the Department of Education, and provides the necessary resources for classroom-implementation. The implementation depends on the collaboration between four partner institutions with the expertise to implement the DE reform and train teachers (El-Hamamsy et al., 2021b). Given the benefits of research-practice partnerships for sustainability (Roesken-Winter et al., 2015, p.7), these institutions partnered with researchers to promote design-based implementation research at all levels of the reform: school culture, school leadership, instructional coaches (ICs), trainers, teachers, pedagogical resources, and students.

Despite the scale of the project, the reform is regional and has placed significant effort on having a PD-program and pedagogical content that are adapted to the regional and school culture by providing the PD-sessions within the schools, with content that is tested in the region, and having tailored long term school-level support in schools with the help of instructional coaches. With these efforts and the attempt to focus on the school-level, the objective was to avoid the “replica trap” (Clarke & Dede, 2009; Coburn, 2003; Lidolf & Pasco, 2020), i.e., repeating “what worked locally, without taking into account local variations in needs and environments” (Clarke & Dede, 2009). Furthermore, since “teachers are better able to sustain change when there are mechanisms in place at multiple levels of the system to support their efforts” (Coburn, 2003, p.5), and to avoid having teachers “revert to their ‘old’ ways entirely after funding and support are withdrawn” (Hubers, 2020; Hubers et al., 2017), instructional coaches are trained to support teachers in each school throughout the DE-PD and in the long-term (see El-Hamamsy et al., 2021band Caneva et al., 2023). The support includes fostering a community of practice within their school (Zehetmeier, 2009; Yadav et al., 2016; Li, 2017), proposing PD-sessions to address school-specific needs (Coburn, 2003), providing technical support (Penuel et al., 2007, p.921), therefore contributing to adapting the reform to the school’s culture (Roesken-Winter et al., 2015).

The corresponding DE-PD was designed to follow teacher education best practices as indicated in (El-Hamamsy et al., 2021b), including those found to contribute to sustaining changes in teacher practices.

Having prolonged PD programs and follow-up support are considered to contribute to the effectiveness and sustainability of PD programs (Penuel et al., 2007; Zehetmeier, 2009; Drits-Esser et al., 2017). The project thus devised a long-term DE-PD program spread over two years. For grade 1-4 teachers, this consisted of 7 daylong PD-sessions (approximately 36 hours), and time with instructional coaches in the schools. The PD-sessions provided balanced theoretical and hands-on practical sessions, a decisive element in PD-settings (Roesken-Winter et al., 2015, p.2). The PD-sessions were separated by several months so that teachers could reflect, appropriate, and test the content in their classrooms, which is considered essential to teacher learning (Roesken-Winter et al., 2015). Continued support, a key element of effective PD (Vaughn et al., 1998), is also provided by instructional coaches during the PD and in the long-term (with an average number of hours per teacher per year ranging from 2.7 to 10.5 depending on the schools, see Caneva et al., 2023).

Curricular alignment, and coherence with the teachers’ context, has also been found to significantly influence the effectiveness of PD programs and increase the likelihood that teachers commit to adopting or adapting the innovation (Penuel et al., 2007; Zehetmeier, 2009; Sullanmaa et al., 2019). In the present case, the curricular alignment of the DE-PD content is ensured by the fact that the curriculum designers collaborated directly with the PD providers, with many individuals having both roles in the project.

Finally, teachers must feel that teaching the discipline is feasible to promote changes in their practice (Drits-Esser et al., 2017; Niederhauser et al., 2018). As providing a focus on classroom practice helps “enhance the chances of successful professional development of primary and secondary education teachers” (Hubers et al., 2020), the content presented during the DE-PD included ready-to-use hands-on unplugged kinaesthetic activities that were linked to existing practices. Teachers could actively test these activities during the DE-PD, and then implement them in their classrooms. These opportunities are considered to positively affect self-efficacy and change in pedagogical beliefs, which facilitates the integration of DE into teacher practices (Ertmer & Ottenbreit-Leftwich, 2010). Furthermore, given the benefits of having teachers plan, enact, and revise curricular units (Penuel et al., 2007, p.931), the teachers were encouraged to adapt the content to their students’ needs and preferences (which is important for classroom management and teachers’ sense of control over the learning environment, Klingner et al., 2001), exchange with their peers, and provide feedback on PD adjustments. The resulting curriculum^2^ and pedagogical resources^3^ are open access and accessible on the Department of Education’s website.

### Analysis methodology

The analysis is carried out in two stages using data acquired from 287 in-service teachers (see Table 1). First, we test the Sustainable Adoption of DE model (SADE, see Section 3) through Structural Equation Modelling (SEM) based on the survey from Table 2 (RQ1). We then investigate whether sustainability has been reached (RQ2) according to the definition proposed in Section 3.1 through a descriptive analysis of the two surveys (see Tables 2 and 3).

**Table 1:** Participants

Survey	N	Grade			Age				Teaching experience				Gender		
		1-2	3-4	Other	N	Min	Max	Mean +/- Std	N	Min	Max	Mean +/- Std	F	M	U
Nov’21	286	131	138	17	286 (U = 13)	21	63	42 +/-11	286 (U = 13)	0	42	18+/-11	270	7	9
Mar’22	284	129	136	19	284 (U = 16)	22	63	41+/-10	284 (U = 16)	1	43	18+/-10	264	9	11

Abbreviations: N = number of responses, U = undisclosed

Please note that i) a grade that is indicated as “other” refers to teachers who do not exclusively teach in either grades 1-2 or grades 3-4 (e.g. they may be teaching across all grades) and ii) approximately 97% of the sample is female which is consistent with the full sample of teachers following the grade 1-4 PD program where 98% are women

**Table 2:** Sustainability Modelling Survey (November 2021, n = 287)

Sustainability Factor	Concept	Original question(s) and scale or supporting literature	Guskey (2000)’s Level	Question adapted to the DE context (and translated from French)	Format
Support (Cronbach’s *α* = 0.79)	Support from school leaders and colleagues (i.e., “extent to which peers reinforce and support use of learning on the job”, Holton et al., 2000)	My colleagues encourage me to use the skills I have learned in training (Learning Transfer System Inventory - LTSI scale, Holton et al., 2000)	Level 3	The school leaders and colleagues support me in integrating Digital Education into my practice	Likert
	Pedagogical and Technical Support (i.e., opportunity to use, Holton et al., 2000)	There are enough human resources available to allow me to use skills acquired in training (LTSI scale, Holton et al., 2000)	Level 3	I have the technical and pedagogical support I need to integrate Digital Education into my practice	Likert
	Time (i.e., “extent to which individuals have the time, energy and mental space in their work lives to make changes required to transfer learning to the job”, Holton et al., 2000)	I have time in my schedule to change the way I do things to fit my new learning (LTSI scale, Holton et al., 2000)	Level 3	I have the time I need to integrate Digital Education into my practices	Likert
	Resources (i.e., “extent to which trainees are provided with or obtain resources and tasks on the job enabling them to use training on the job”, Holton et al., 2000)	The resources I need to use what I learned will be available to me after training (LTSI scale, Holton et al., 2000)	Level 3	I have the material resources I need to integrate Digital Education into my practice	Likert
Perceived ease of use (Cronbach’s *α* = 0.8)	Adequacy of the content with respect to teachers’ practices (i.e., compatibility with prior practices or perceived content validity which refers to the extent to which trainees judge training content to accurately reflect job requirements, LTSI scale, Holton et al., 2000); *M* = 1.03 ± 1.60, Cronbach’s *α* = 0.75)	What is taught in training closely matches my job requirements (LTSI scale, Holton et al., 2000)	Level 2	The following types of activities are adapted to my practice:	Likert
				a) unplugged CS activities without robots or tablets (e.g. robot game, sorting machine, networks, pixel screen)	
				b) unplugged CS robotic activities (e.g. Bluebot and pre-programmed Thymio)	
				c) plugged CS robotic activities (e.g. Thymio visual programming)	
				d) plugged CS non robotic activities (e.g. Scratch)	
				e) ICT activities (e.g. BookCreator, Clips, Keynote)	
				f) Dialogues around digital literacy in the classroom (e.g. sharing, saying no, advertising)	
				g) Acquiring digital literacy by reading a book (e.g. Oscar and Zoe, Loupé, Pffff)	
	Understanding	After the training, I know substantially more about the training contents than before. I learned a lot of new things in the training (Questionnaire for Professional Training Evaluation, Grohmann & Kauffeld, 2013)	Level 2	I understand the Digital Education content seen in the PD program	Likert
	Community of practice	(Eickelmann, 2013; Redmond et al., 2021; Wise, 2021; Stoetzel & Shedrow, 2020; Li, 2017)	Level 3	I exchange ideas and resources related to Digital Education with my colleagues	Likert
	Self-efficacy (i.e., performance self-efficacy, or “an individual’s general belief that they are able to change their performance when they want to”, Holton et al., 2000).	I am confident in my ability to use newly learned skills on the job (LTSI, Holton et al., 2000)	Level 2	I am able to integrate Digital Education into my practice	Likert
	Appropriation (i.e., Technological pedagogical content knowledge)	I can select technologies to use in my classroom that enhance what I teach, how I teach, and what students learn (TPACK.xs survey, Schmid et al., 2020)	Level 2	I can see how to complete, extend, and integrate variants of the resources at my disposal according to my educational objectives	Likert
Perceived usefulness (Cronbach’s *α* = 0.73 )	Useful to Teach	(King & He, 2006; El-Hamamsy et al., 2021a; El-Hamamsy et al., 2022a)	Level 5	I think that teaching Digital Education is useful	Likert
	Utility of teaching with ICT	The training is very beneficial to my work. Participation in this kind of training is very useful for my job (Questionnaire for Professional Training Evaluation, Grohmann & Kauffeld, 2013) ; I believe the training will help me do my current job better (Teo et al., 2011)	Level 5	I think the use of digital technologies makes me more effective in my teaching.	Likert
	Teacher & student learning benefits	The training is very beneficial to my work. Participation in this kind of training is very useful for my job (Questionnaire for Professional Training Evaluation, Grohmann & Kauffeld, 2013); I believe the training will help me do my current job better (Teo et al., 2011)	Level 5	I think that teaching Digital Education brings me benefits (e.g. feeling of competence, student learning...)	Likert
	Costly to teach	The use of computer technology in the classroom is too costly in terms of resources, time and effort (Technology Implementation Questionnaire, Wozney et al., 2006)	Level 5	I think that teaching Digital Education costs me (e.g. time, effort...)	Likert
Actual usage	Adoption quantity	(King & He, 2006; El-Hamamsy et al., 2021a; El-Hamamsy et al., 2022a)	Level 4	During the 2020-2021 academic year, which of the following activities did you or your instructional coach implement with your students? (25 pedagogical activities and a none option)	Checkboxes
	Adoption frequency	(King & He, 2006; El-Hamamsy et al., 2021a; El-Hamamsy et al., 2022a)	Level 4	How many periods did you dedicate to the Digital Education activities that you taught? (up to 25 values)	Numeric

Likert scale items are evaluated on a 7-point scale ranging from -3 (completely disagree) to + 3 (completely agree)

**Table 3:** Follow-up sustainability survey (March 2022, n = 284)

Sustainability Factor	Concept	Supporting literature	Guskey (2000)’s Level	Question	Format
Support	Support needed to continue to teach Digital Education (Cronbach’s *α* = 0.88)	(Eickelmann, 2013; Redmond et al., 2021; Wise, 2021; Stoetzel & Shedrow, 2020)	Level 3	To continue to integrate Digital Education in my classrooms, I need	Likert
				a) somebody to come in and teach the activities (the IC teaches the activities alone)	
				b) to co-teach (the activities is taught jointly with the IC)	
				c) co-planning (the activities is prepared in advance with the IC, and I teach it alone in class)	
				d) technical management assistance with the IC; e) training workshops with the ICs	
				f) in-service training focused on the appropriation of digital tools	
				g) in-service training focused on the integration of resources in the various disciplines	
				h) in-service training focused on classroom management	
	Time (a part of the Personal Capacity for transfer, i.e., “the extent to which individuals have the time, energy, and mental space in their work lives to make changes required to transfer learning to the job”, (Holton et al., 2000); Cronbach’s *α* = 0.94)	(Guskey, 2000; Karsenti & Bugmann, 2018; Redmond et al., 2021; Penuel et al., 2007)	Level 3	To teach Digital Education today I think I have / had enough:	Likert
				a) Training time	
				b) Time to appropriate the basic concepts	
				c) Time to appropriate the tools	
				d) Time to appropriate the student content	
				e) Time to prepare the lessons	
				f) Time in class to integrate the student content	
				g) Time for exchanges with colleagues	
				h) Time for exchanges with the ICs	
Perceived utility	Student Learning benefits	(Tikkanen et al., 2020; Vaughn et al., 1998; Klingner et al., 2001; Li, 2017; Jamaludin & Hung, 2016)	Level 5	I think my students are progressing thanks to the proposed resources	Likert
	Costs (a part of the Personal Capacity for transfer, i.e., “the extent to which individuals have the time, energy, and mental space in their work lives to make changes required to transfer learning to the job”, (Holton et al., 2000); Cronbach’s *α* = 0.77)	(Wigfield & Cambria, 2010; Cèbe & Goigoux, 2018; Klingner et al., 2001; Guskey, 2000; Tikkanen et al., 2020; Hubers, 2020)	Level 5	Implementing the proposed resources is too cognitively demanding	Likert
			Level 5	Implementing the proposed resources requires too much work	Likert
			Level 5	Implementing the proposed resources requires too much emotional investment	Likert
Actual usage	Adoption quantity	(King & He, 2006; El-Hamamsy et al., 2021a; El-Hamamsy et al., 2022a)	Level 4	During the 2021-2022 academic year, which of the following activities did you or your instructional coach implement with your students? (25 pedagogical activities and a none option)	Checkboxes
	Adoption frequency	(King & He, 2006; El-Hamamsy et al., 2021a; El-Hamamsy et al., 2022a)	Level 4	How many periods did you dedicate to the activities that you taught? (up to 25 values)	Numeric

The Likert scale items are evaluated on a 7-point scale ranging from -3 (completely disagree) to + 3 (completely agree)

More specifically for RQ1, SEM is applied to the survey data in Table 2 based on the SADE model to determine to what extent the structural model fits the data based on the fit indices (see Table 4). However, prior to applying SEM, the measurement model must be validated through Confirmatory Factor Analysis (CFA, Hamid et al., 2017). This validation requires ensuring that the global and local fit indices in Table 4 are met for the CFA, in addition to verifying the criteria in Table 5. Since “it is generally recommended to use [...] robust SEM methods, rather than directly deleting outliers and influential observations” (Lai & Zhang, 2017), all modelling (CFA, SEM) is done using robust estimators. The modelling is carried out in R (version 4.2.1, Core Team R, 2019) with lavaan (version 0.6-11, Rosseel, 2012), semTools (version 0.5-6, Jorgensen et al., 2022), semTable (version 1.8, Johnson & Kite, 2020), psych (version 2.2.5, Revelle, 2022), and semPlot (version 1.1.5, Epskamp, 2022).

**Table 4:** Global and local fit indices for confirmatory factor analysis and structural equation modelling

Metric	References	Explanation	Recommended Threshold
The *χ*^2^ statistic	(Alavi et al., 2020; Prudon, 2015)	Tests the null hypothesis that the predicted model and observed data are equal based on the sample and covariance matrices (which is a required condition), but is sensitive to sample size, with larger samples decreasing the p-value	$p_{\chi ^{2}}>0.05$
The ratio between the *χ*^2^ statistic and the degrees of freedom *χ*^2^/*df*	(Kyriazos, 2018)	Tests the null hypothesis that the predicted model and observed data are equal but is less sensitive to sample size	*χ*^2^/*df* ≤ 5 for acceptable fit, *χ*^2^/*df* ≤ 3 for good fit
The standardised root mean square residual (SRMR)	(Xia & Yang, 2019; Hu & Bentler, 1999)	The standardised difference between the observed correlation and the model implied correlation matrix	*SRMR* < 0.08
The Root Mean Square Error of Approximation (RMSEA)	(Xia & Yang, 2019; Hu & Bentler, 1999; Chen et al., 2008)	An absolute measure of fit which indicates how far the model is from a perfect model.	*RMSEA* < .08 for acceptable fit, *RMSEA* < .06 for good fit
The comparative fit index (CFI)	(Xia & Yang, 2019; Byrne, 1994; Schumacker & Lomax, 2004)	An incremental fit index which compares the fit of the target model to the fit of a baseline model which has the worst fit using the using the difference between *χ*^2^ statistic and the number of degrees of freedom	*CFI* > 0.9 for acceptable fit, *CFI* > 0.9 for good fit
The Tucker Lewis index (TLI)	(Xia & Yang, 2019; Byrne, 1994; Schumacker & Lomax, 2004)	An incremental fit index which compares the fit of the target model to the fit of a baseline model which has the worst fit using the using the ratio between the *χ*^2^ statistic and the number of degrees of freedom	*TLI* > 0.9 for acceptable fit, *TLI* > 0.9 for good fit

**Table 5:** Measurement model validation criteria through CFA

Metric	Explanation and References	Recommended
		Threshold
Sample size	The sample size needs to be sufficient to be estimate all the model’s parameters. The recommended ratio between observations and number of parameters to be estimated is generally 10:1 (Ockey & Choi, 2015), with 5:1 being the point at which estimates may become unstable (Suhr, 2006; Bentler & Chou, 1987). However, researchers consider that such ratios are not necessarily valid (Ockey & Choi, 2015). Researchers thus increasingly recommend sample size calculators (Morrison et al., 2017) such as (Soper, 2022)’s sample size calculation tool for SEMs which is based on (Cohen, 1988; Christopher Westland, 2010)	Sample size sufficient to detect medium effect sizes
Skew and Kurtosis	The individual items’ skew and kurtosis should be within the recommended ranges for normality (Ockey & Choi, 2015)	*abs*(*skew*) < 3, *abs*(*kurtosis*) < 10
Internal consistency of the sub-scales	Internal consistency of the latent factors’ sub-scales must be sufficiently high to be a reliable measure of the corresponding latent factor and can be established through Cronbach’s *α* and the Composite Reliability (CR) for all sub-scales (Cortina, 1993; Hamid et al., 2017)	*α* in [0.7 − 0.9], *CR* > 0.7
Convergent Validity	Extent to which the items are representative of the latent variable they intend to measure (Lee & Louis, 2019; Fornell & Larcker, 1981), metrics such as the Average Variance Extracted (AVE) can be used in conjunction with the Composite Reliability (CR)	*AV* *E* > 0.5 for good fit, and *AV* *E* > 0.4 if *CR* > 0.6 for adequate fit
Discriminant validity	An indication of the extent to which the different latent factors are unrelated (Hamid et al., 2017) which can be established through (Rönkkö & Cho, 2022)’s discriminant validity test	Correlations between latent factors < 0.9
Fit indices	See global and local indices Table 4	
CFA Factor loadings	Factor loadings indicate the extent to which the observed variables explain the variance of the associated latent variable. For a given factor loading, the amount of variance explained is equal to the square of the loading value (e.g. a loading of 0.5 will explain 0.5^2^ = 0.25, i.e., 25% of the variance of the associated latent factor)	Significant loading with standardised value above 0.3

## Results

### RQ1: What factors significantly influence the Sustainability of the reform?

#### Verifying the applicability of structural equation modelling

##### Sample size and missing data

Given the importance of having a sufficient sample for such types of analyses, we verify under which conditions we may be able to apply SEM in the present context. At most, of the 287 observations, there are 10 missing values for a given indicator (see Table 6). As the model requires estimating 31 free parameters, the ratio between the observations and parameters to be estimated is around 9:1 and close to the recommended 10:1 ratio. Soper (2022)’s sample size calculation tool indicates that with a desired statistical power of 0.8, 3 latent variables, 14 observed variables, and a probability level *α*= 0.05 the minimum effect that can be detected is approximately 0.21. We are thus above the threshold required to conclude for medium effect sizes.

**Table 6:** Reliablity of the scale, and descriptive statistics of the items in the measurement model

	Count	Missing	Mean ([-3; + 3])	SD	Skew	Kurtosis
(1) Perceived usefulness vs. cost	281	6.0	0.09	1.00	-0.10	0.54
(Cronbach’s *α* = 0.73, *AV* *E* = 0.471, *CR* = 0.762)						
Useful to teach	278	9	1.10	1.17	-0.40	0.47
Not costly to teach (reversed)	281	6	-1.24	1.35	0.69	0.45
Teacher & student learning	281	6	0.40	1.39	-0.39	0.20
Utility of teaching with ICT	281	6	0.13	1.46	-0.07	-0.56
(2) Support	281	6	0.68	0.99	-0.23	0.39
(Cronbach’s *α* = 0.79, *AV* *E* = 0.502, *CR* = 0.805)						
Sufficient Resources	281	6	0.78	1.29	-0.68	0.53
Sufficient Time	280	7	-0.28	1.41	0.06	-0.54
Support from the hierarchy and colleagues	279	8	1.27	1.06	-0.18	0.02
Technological and pedagogical support	280	7	0.98	1.26	-0.48	0.41
(3) Perceived ease of use	281	6	0.85	0.89	-0.10	-0.12
(Cronbach’s *α* = 0.8, *AV* *E* = 0.506, *CR* = 0.814)						
Understanding	277	10	1.68	0.94	-0.33	-0.18
Community of practice	281	6	0.47	1.37	-0.28	-0.52
Appropriation	280	7	0.28	1.39	-0.31	-0.04
Adequacy of the content	281	6	1.09	0.83	-0.28	0.30
Self-efficacy	281	6	0.73	1.31	-0.58	0.55

##### Reliability and descriptive statistics

The indicators of the measurement model are provided in Table 6 with their descriptive statistics and reliability indicators. The skew, kurtosis, Cronbach’s *α* and Composite Reliability are all within their desired ranges, supporting the reliability of the measurement model.

##### Model Fit

The robust fit indices are acceptable (see the criteria in Table 4) with *χ*^2^(62)= 128, *p*< 0.001, *χ*^2^/*df*< 2, *CFI*= 0.922, *TLI*= 0.902, *RMSEA*= 0.063, *RMSEA* 90*%ci*=[0.047;0.079], *SRMR*= 0.054.

##### Indicator Reliability

Table 7 shows that the standardised factor loadings exceed 0.3 and are significant (*p* < 0.001) for all indicators and support the reliability of the indicators.

**Table 7:** Measurement model factor loadings established through confirmatory factor analysis

Latent factor	Variable	Estimate	Std. Err.	Z	p	R-Square	Standardised
							Estimate
Support	Sufficient Time	1.014	0.098	10.330	0.000	0.512	0.715
	Sufficient Resources	0.899	0.087	10.300	0.000	0.487	0.698
	Technological and pedagogical support	0.925	0.074	12.551	0.000	0.540	0.735
	Support from the hierarchy and colleagues	0.715	0.062	11.559	0.000	0.454	0.673
Perceived usefulness vs. cost	Useful to teach	0.799	0.072	11.057	0.000	0.464	0.681
	Teacher & student learning	1.120	0.082	13.622	0.000	0.656	0.810
	Utility of teaching with ICT	1.154	0.074	15.689	0.000	0.618	0.786
	Not costly to teach	0.433	0.104	4.177	0.000	0.104	0.322
Perceived ease of use	Adequacy of the content	0.526	0.049	10.756	0.000	0.392	0.626
	Understanding	0.527	0.053	9.964	0.000	0.311	0.558
	Appropriation	1.085	0.078	13.950	0.000	0.595	0.771
	Self-efficacy	1.026	0.076	13.507	0.000	0.602	0.776
	Community of practice	0.928	0.079	11.681	0.000	0.459	0.677

##### Convergent Validity

The AVE and CR are provided in Table 6. The values meet the requirements posed in Section 4.2 and support the convergent validity of the CFA.

##### Discriminant Validity

**Fig. 3:**
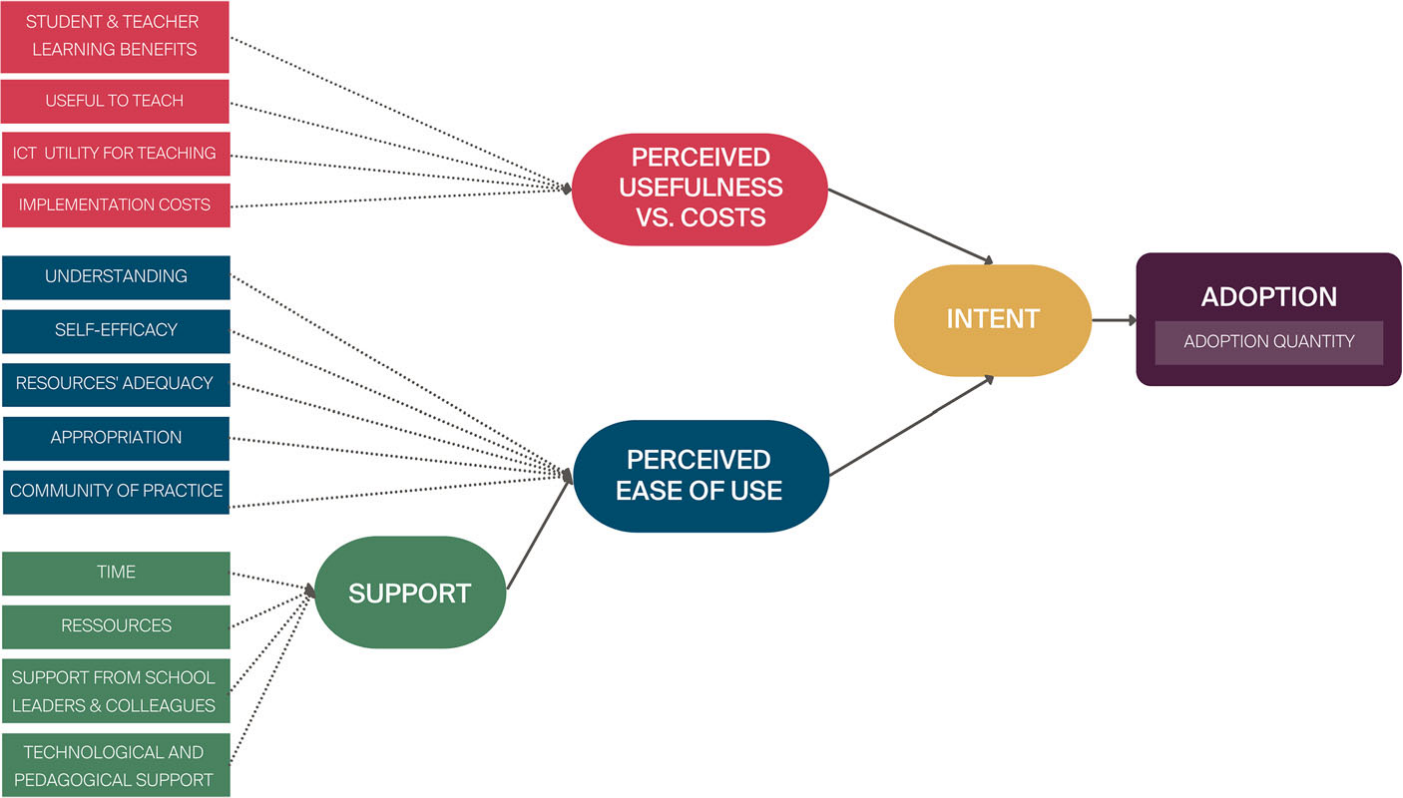
Second Order SADE Model Introducing a Second Order Latent Variable Referred to as Intent

Rönkkö and Cho (2022)’s discriminant validity test indicated that the comparison between pairs of latent factors yields significant *χ*^2^ tests (*p* < 0.00), except between the utility and ease of use factors which were highly correlated (> 0.9). As these two factors differ conceptually, rather than combining them into a single latent variable, we consider a second-order model (see Fig. 3) where Perceived Ease of Use and Perceived Usefulness impact Adoption Quantity through a mediating latent variable (in this case Intent as in the TAM models, King and He (2006). As the second-order factor (i.e., Intent) will only have two indicators, in order to identify it, we constrain the factor loadings of Perceived Ease of Use and Perceived Usefulness to be equal. As such, the second-order model is equivalent to a first-order model where utility and ease of use would be combined in a single latent factor.

#### Modelling the Sustainability of the adoption of digital education

As the dependent variable (number of activities adopted) follows a non-normal distribution (Shapiro-Wilk *W*= 0.9579, *p*< 0.0001; Anderson-Darling *A*= 9.456, *p*< 0.0001), the structural equation model is estimated using a robust diagonally weighted least squares estimator to be robust to missing values and outliers (Lai & Zhang, 2017),. The overall fit is acceptable (*χ*^2^(75)= 144, *p*< 0.001; *χ*^2^/*df*< 2; *CFI*= 0.921; *TLI*= 0.904; *RMSEA*= 0.059; RMSEA 90*%ci*=[0.044;0.073]; *SRMR*= 0.054).

**Fig. 4:**
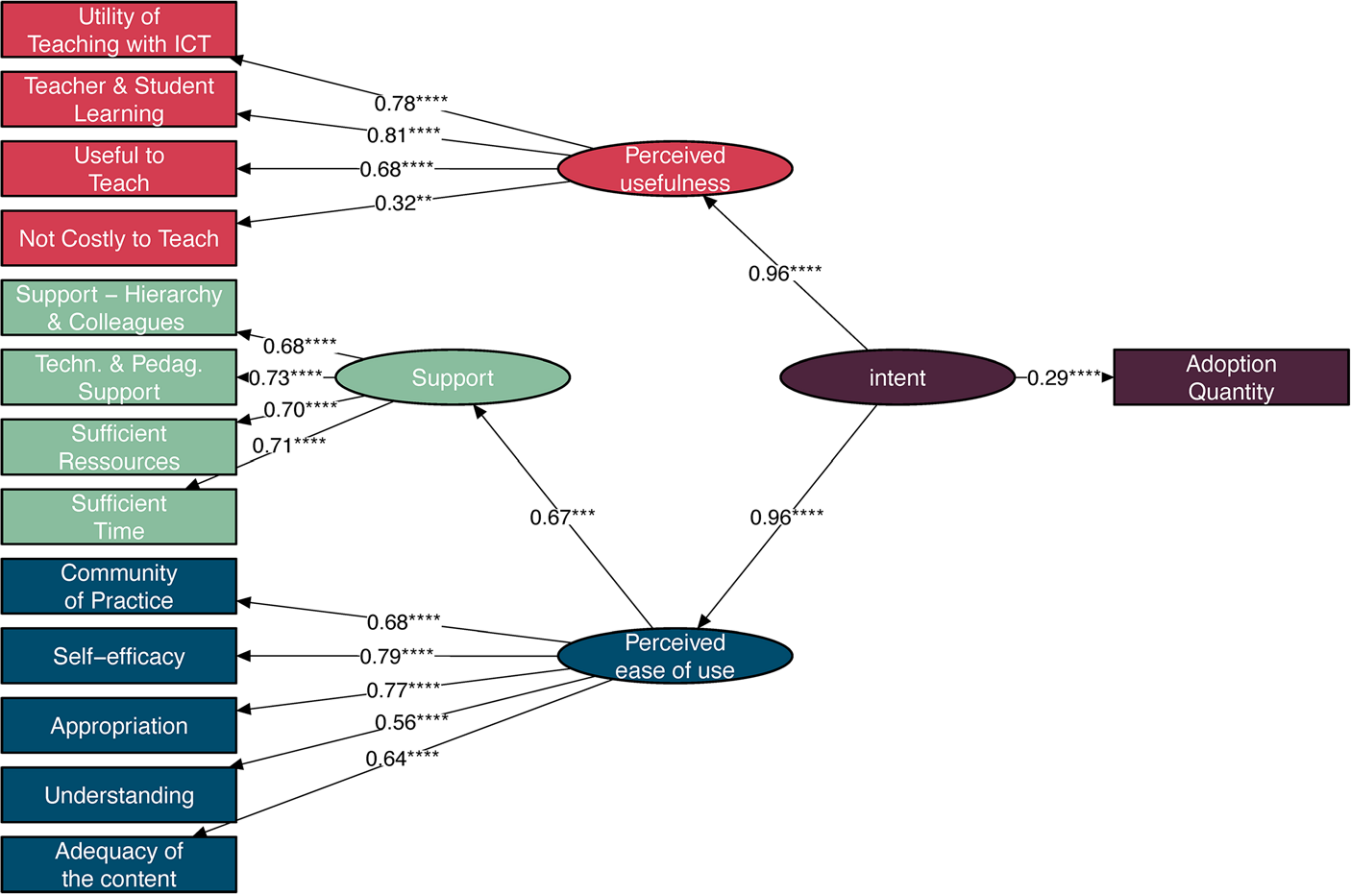
Second order SADE model path diagram

The standardised factor loadings, regression parameters, direct effects and indirect effects are provided in Table 8, and the resulting path model in Fig. 4. All the indicators are significantly and positively correlated with their respective latent variables (*p* < .0001) with high levels of correlation (*β* > 0.558), with the exception of perceiving teaching DE to be costly which negatively impacts the perceived usefulness vs. cost latent factor (*β* = − 0.32, *p* = 0.001). The paths between latent variables are also significant and highly correlated (*β* < 0.67, *p* < 0.0001). Finally, the effect of the intent latent variable on Adoption quantity is significant (*p* < 0.0001) and in the low-medium range (0.285).

**Table 8:** Structural equation model factor loadings, regression parameters, and indirect effects

Latent factor	Variable	Estimate	Std. Err.	Z	p	R-Square	Standardised
							Estimate *β*
Support	Sufficient Time	0.750	0.096	7.803	0.000	0.504	0.710
	Sufficient Resources	0.673	0.089	7.570	0.000	0.492	0.701
	Technological and pedagogical support	0.690	0.079	8.692	0.000	0.540	0.735
	Support from the hierarchy and colleagues	0.536	0.068	7.927	0.000	0.458	0.677
Perceived usefulness vs. cost	Useful to teach	0.212	0.050	4.265	0.000	0.457	0.676
	Teacher & student learning	0.299	0.066	4.530	0.000	0.651	0.807
	Utility of teaching with ICT	0.306	0.069	4.447	0.000	0.608	0.779
	Not costly to teach (reversed)	0.114	0.036	3.191	0.001	0.101	0.318
Perceived ease of use	Adequacy of the content	0.143	0.033	4.399	0.000	0.406	0.638
	Understanding	0.141	0.032	4.463	0.000	0.312	0.558
	Appropriation	0.291	0.064	4.513	0.000	0.596	0.772
	Self-efficacy	0.278	0.060	4.635	0.000	0.617	0.786
	Community of practice	0.251	0.057	4.401	0.000	0.467	0.683
	Support	0.239	0.062	3.841	0.000	0.444	0.667
Intent	Perceived ease of use	3.599	0.806	4.467	0.000	0.964	0.964
	Perceived usefulness vs. cost	3.599	0.806	4.467	0.000	0.964	0.964
Regression	Latent factor	Estimate	Std. Err.	Z	p	R-Square	Standardised Estimate
Adoption quantity	$\sim $ Intent	0.702	0.175	4.018	0.000	0.081	0.285
Indirect Effects on Adoption	Path	Estimate	Std. Err.	Z	p	R-Square	Standardised Estimate
support − >	ease− > intent − > adoption	0.605	0.190	3.188	0.001	0.444	0.183
ease − >	intent − > adoption	2.525	0.863	2.927	0.003	0.928	0.275
utility− >	intent − > adoption	2.525	0.863	2.927	0.003	0.928	0.275

The results thus support the foundation of the second-order SADE model, from which we extract four key findings.

First, perceived ease of use (understanding and appropriating the content) and perceived usefulness vs. costs (benefits of teaching DE) impact adoption quantity through a mediating intent variable with a standardised total effect of *β* = 0.275 each on adoption quantity. Contrary to the model proposed in Section 3, this mediation effect would indicate the importance of considering an intrinsic motivation component, which may be affected by the way the content is perceived in terms of ease of use and utility, as in TAM models (King & He, 2006). However, both ease of use and utility appear to be quite intertwined at this stage of the program, as indicated by the CFA and the high correlation between these two latent factors.

Second, perceived ease of use is moderated by external factors which provide teachers with the means and support required to introduce the discipline into their practice, which contributes to a total standardised effect of *β* = 0.183 on adoption quantity. Although teachers have had three years to introduce the new curriculum into their practice, the need for external support and its impact on adoption remains high. Whether this has decreased with respect to the start of the program or has stabilised remains an open question. However, the implication is clear: support in the schools, whether from colleagues or school leaders, or from a pedagogical or technical standpoint, must continue over time and not decrease after the PD program ends. Their need should be re-evaluated once sustainability has been reached to determine to what extent these are needed beyond the point where teachers’ practices have stabilised.

Third, all the indicators have significant paths, implying that all these factors must be considered both at the start of Digital Education curricular reforms, and sustained until a stabilisation of adoption has been reached. Interestingly, the results also indicate that the benefits outweigh the costs in teachers’ decisions to adopt the discipline at this stage of the program (in the fourth year, i.e., more than a year after no longer being in contact with the two-year DE-PD program). With teachers expressing certain doubts about the benefits of teaching the discipline for them and for their students, it would thus appear essential to gain further insight into the students’ perspective, not only to have a complete evaluation of the PD program, but also to give teachers direct insight into the learning benefits of teaching the discipline.

Finally, given the explained variance in adoption (*R*^2^ = 0.081), additional factors must be considered to gain a more in-depth understanding of teachers’ decisions to adopt DE pedagogical content and the extent to which they do so.

### RQ2: Has sustainability of the digital education curricular reform been reached?

#### Adoption of DE pedagogical content over time

**Fig. 5:**
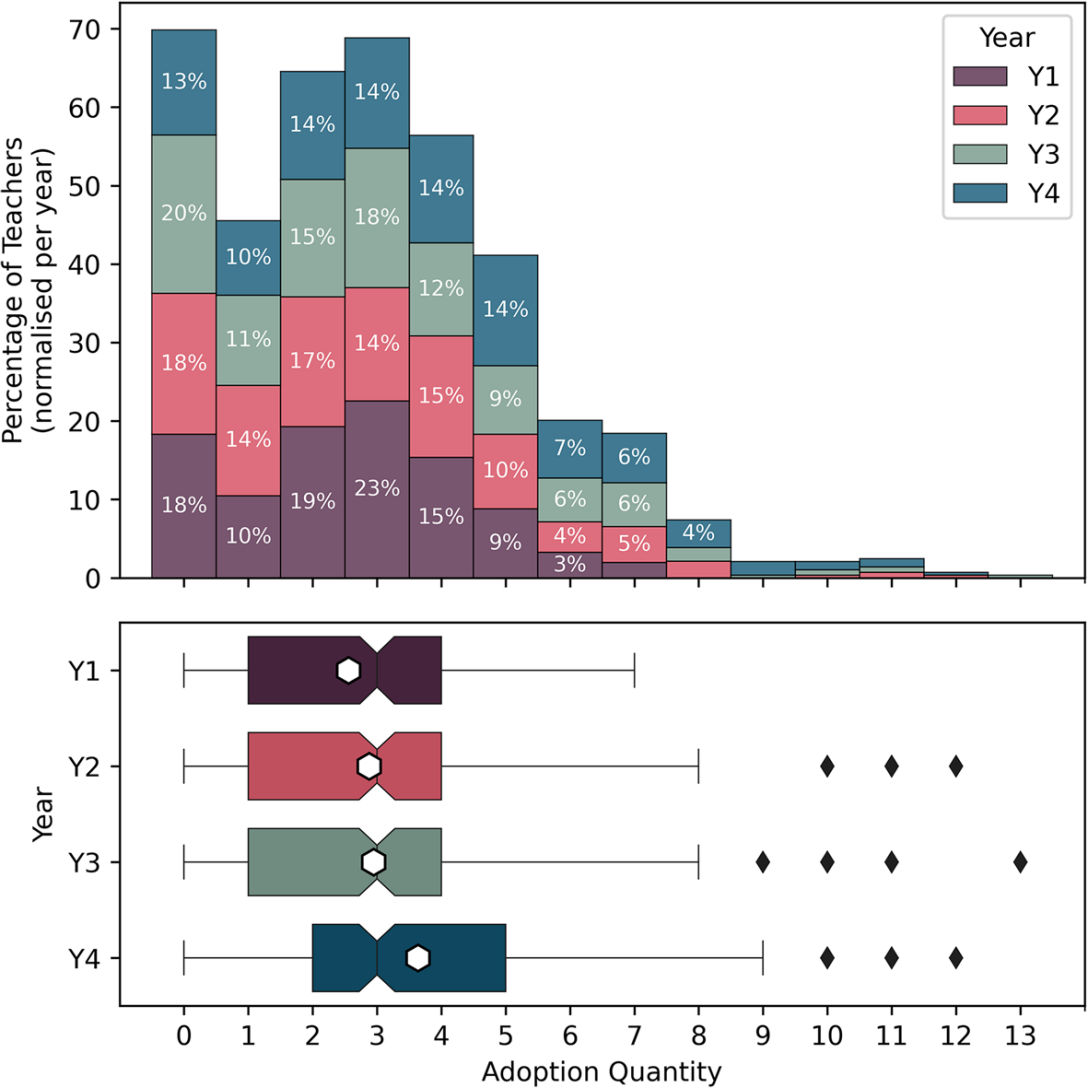
Evolution of the adoption of Digital Education pedagogical activities over the four year period. The first and second years correspond to the start of the curricular reform and the duration of the DE-PD program. The teachers were no longer in contact with PD providers in the third year

Previous studies with the cohort of teachers included in the present study showed high satisfaction with the DE-PD program, with an overall satisfaction of *M* = 3.58 ± 0.58 for the Computer Science portion of the DE-PD (year 1) and *M* = 3.17 ± 0.54 for the ICT and Digital citizenship portion of the DE-PD (year 2) on a 4-Point Likert scale (i.e. between 1 and 4). These results are believed to have contributed to positive uptake of the DE pedagogical content over the first two years of the reform (El-Hamamsy et al., 2021b). To complement these results, we consider the number of DE activities teachers taught over the 4 years of the DE curricular reform (see Fig. 5). The adoption rate (i.e., proportion of teachers adopting at least one activity) is greater than 80%, reaching 87% in the fourth year of the curriculum. These results are promising considering that approximately 10% of teachers do not implement changes (Gersten et al., 2000), indicating that it is unlikely that more teachers would teach DE. However, it is also important to consider the amount taught by the teachers. On average, teachers teach 3 DE activities per year, which appears to be steadily increasing. The difference between the years is significant (Kruskal Wallis^4^*H* = 25.7, *p* < 0.0001), with Dunn’s post-hoc test indicating that the difference is significant between adoption in years 1-3 and adoption in year 4 (see Table 9). Although the teachers’ practices have not yet stabilised, the results appear promising in terms of sustainability. There are, however, other indicators that must be accounted for, which we explore in the following subsections.

**Table 9:** Yearly adoption rates and significant differences between the number of activities adopted each year established using Dunn’s post-hoc test with Benjamini-Hochberg p-value correction for multiple comparisons

	Adoption rate	Year 1	Year 2	Year 3	Year 4
Year 1 (n = 306)	82%		p = 0.33509, D = 0.16	p = 0.31481, D = 0.18	p = 1e-05, D = 0.49
Year 2 (n = 284)	82%	p = 0.33509, D = 0.16		p = 0.86827, D = 0.03	p = 0.00077, D = 0.31
Year 3 (n = 287)	80%	p = 0.31481, D = 0.18	p = 0.86827, D = 0.03		p = 0.00094, D = 0.27
Year 4 (n = 284)	87%	p = 1e-05, D = 0.49	p = 0.00077, D = 0.31	p = 0.00094, D = 0.27	

#### Perceived ease of use

**Fig. 6:**
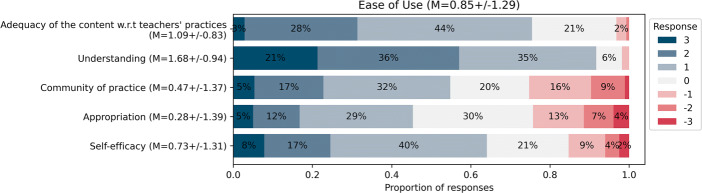
Responses for the SADE models’ Ease of Use dimension (November 2021, n = 287)

When considering only strictly positive responses (i.e., ≥ 1 on the 7-Point Likert scale, see Fig. 6), 89*%* of teachers consider that they have understood the underlying DE concepts, with 74*%* on average considering that the pedagogical content is adequate with respect to their practice. However, these proportions are lower when it comes to self-efficacy, since 63*%* teachers consider being able to teach the content. Furthermore, only 54*%* exchange ideas and resources with their colleagues and just 44*%* consider that they are able to adapt and appropriate the content to their educational objectives (second stage of instrumentation, Trouche, 2005). The self-efficacy responses appear to be consistent with the proportion of teachers who have adopted 2 or more activities in the third year (68*%*). This could be indicative of the fact that teachers need to have tested the content to feel confident in their capacity to teach it (Ertmer & Ottenbreit-Leftwich, 2010).

#### Support

**Fig. 7:**
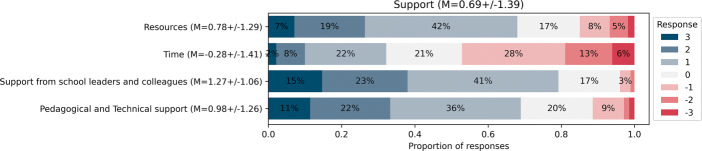
Responses for the SADE models’ Support dimension (November 2021, n = 287)

**Fig. 8:**
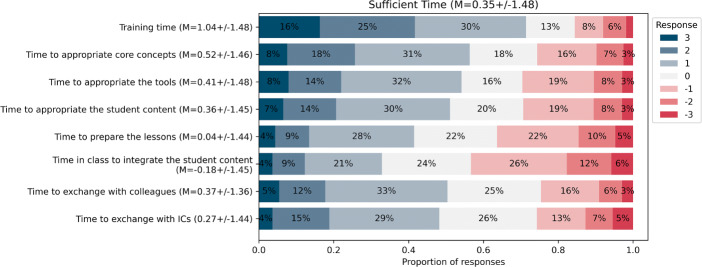
Teachers’ perception of having received enough time along multiple dimensions (i.e. training, appropriation, training, exchanges with colleagues, March 2022, n = 284)

**Fig. 9:**
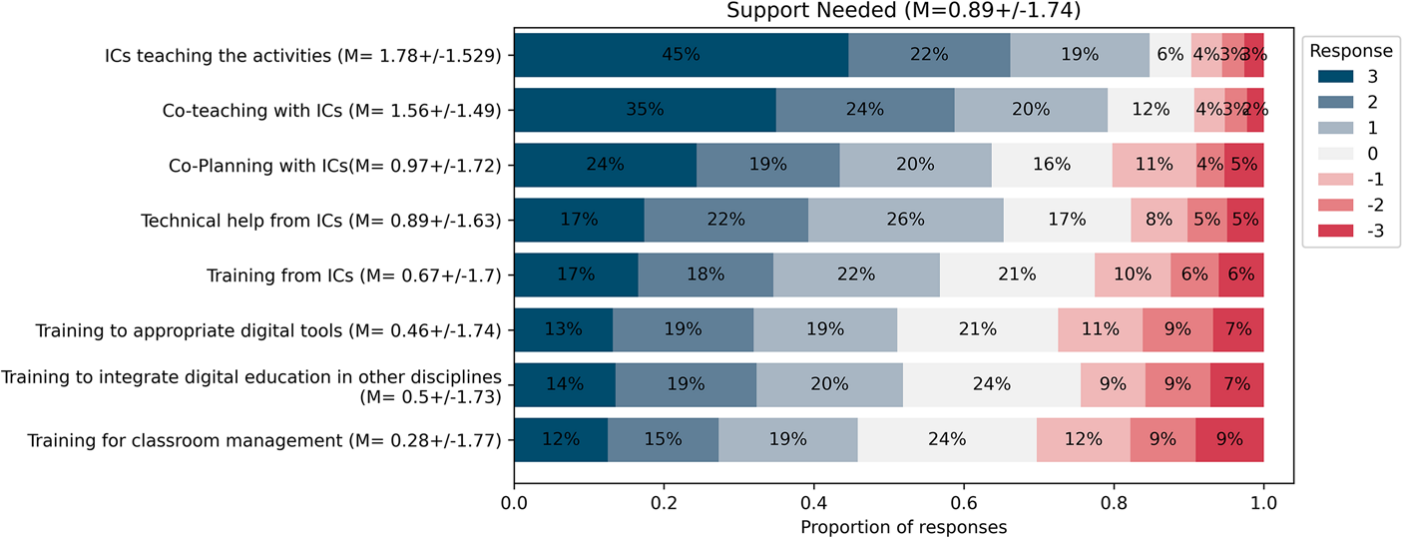
Teachers’ reported needs to continue to integrate Digital Education into their practice (March 2022, n = 284)

As shown in Fig. 7, and considering only strictly positive responses (i.e., ≥ 1) on the 7-point Likert scale, teachers consider that they have the support they need from the school leadership and their colleagues (77*%*), the technological and pedagogical support (67*%*) and access to resources (67*%*) to teach the discipline, which is consistent with the proportion of teachers who have adopted two or more activities in the third year (68*%*). However, one main issue appears: teachers disagree with having the time they need to integrate the discipline into their practice (just 30*%* consider that they have sufficient time). A more in-depth investigation (see Fig. 8) reveals that teachers believe they have received enough training time (69*%*) but require more appropriation time (45 − 50*%*), and time to exchange with colleagues (51 − 53*%*), with the main barrier being a lack of time to prepare the lectures (60*%*) and integrate the content into their lesson time (68*%*). Unfortunately, this lack of time with respect to lesson time appears despite the DE content being part of the mandatory curriculum. In addition to time, when asking the teachers what they would need to continue to integrate the discipline into their practice in the long term (see Fig. 9), the most prominent need was to delegate and have instructional coaches teach the activities in their classroom for them (80%). This is surprising considering that most teachers believe they had enough training time (69%), and are confident that they can teach the content (63%).

#### Perceived usefulness vs. costs

##### Costs and efforts required to integrate the discipline

**Fig. 10:**
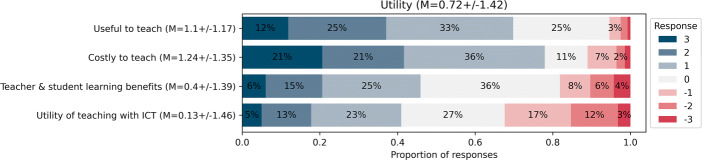
Responses for the SADE models’ Utility dimension (November 2021, n = 287)

**Fig. 11:**
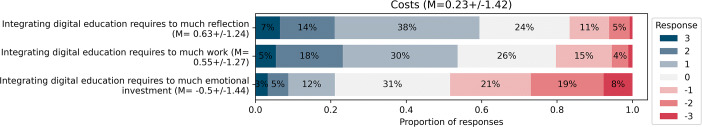
Teachers’ perception of the costs related to integrating Digital Education into their practice (March 2022, n = 284)

**Fig. 12:**
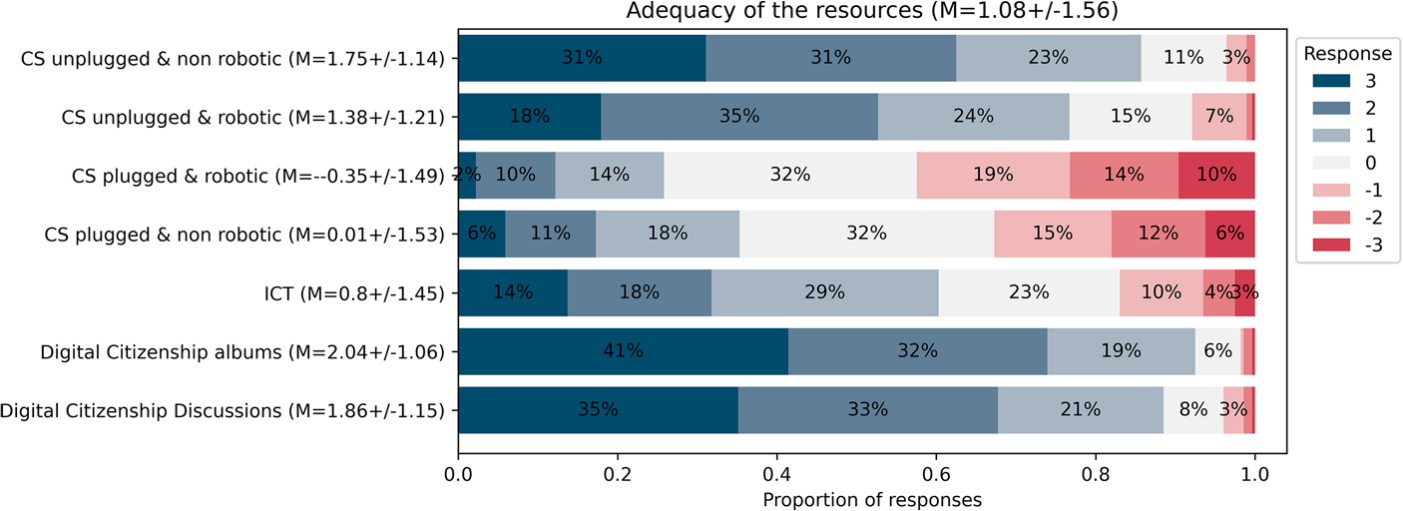
Teachers’ perception of the adequacy of the content with respect to their practice according to the activity type (November 2021, n = 287)

**Fig. 13:**
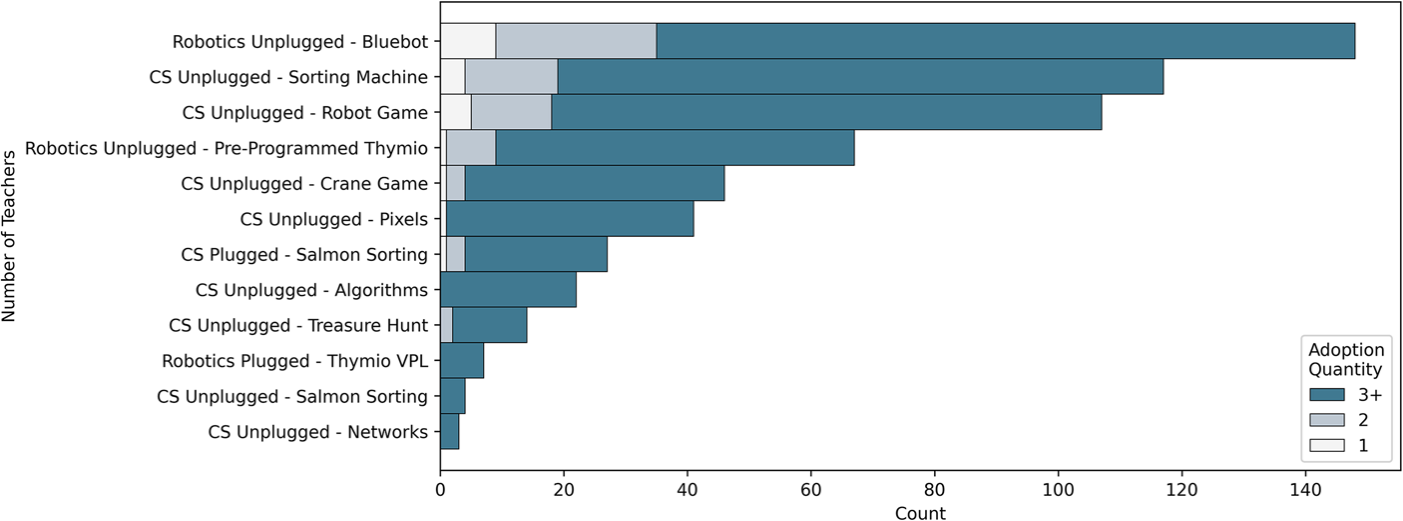
Computer Science activities adopted with respect to the activity type in Year 3 (November 2021, n = 287)

Although teachers believe that they have received sufficient training time (see Fig. 8), find the resources mainly adequate (see Fig. 6), and believe that they have the support they need (see Fig. 7) they still deem it costly to integrate the content into their practice 3 years into the curricular reform (76*%*, see Fig. 10). This is not in terms of emotional investment, but in terms of amount of reflexion and work required: just 16*%* of teachers find that it does not require too much reflexion and 20*%* find that it does not require too much work to teach DE (see Fig. 11). However, the efforts required likely depend on the type of activities since the content differs in terms of perceived teacher adequacy (see Fig. 12). The more digital tools are required to teach the content (e.g. using a tablet or programming a robot), the less these are considered adequate with respect to teachers’ practice . The activities that are considered the most adequate are thus unplugged (whether robotic or not) and do not require the use of tablets or computers (i.e., CS unplugged and digital citizenship activities). The least adequate activities are those that require using a tablet or computer, with the culmination being those employing both a tablet (or computer) and a robot. Drawing from the example of Computer Science content for this group of teachers which was entirely introduced in the first year of the DE-PD, the number of teachers adopting each activity is shown in Fig. 13. Teachers who adopted just one activity opted mainly for unplugged activities (e.g. Bluebot, Sorting Machine, Robot Game) and did not engage in activities which involved programming and thus required a tablet or computer to do so (despite having the material resources to do so). Furthermore, the activity that involved just programming a virtual agent (i.e., Scratch) was adopted by more teachers than the one that involved programming a robot (i.e., Thymio VPL).

##### Perceived usefulness of integrating the discipline into their practice

As Figs. 10 and 11 show, despite teachers considering it useful to teach DE (68*%*), they do not appear to be convinced that they or their students are benefiting (45*%* responding positively). Only 35*%* believe that their students are learning as a result of teaching DE, with 14*%* responding negatively, and the majority (51*%*) agreeing or disagreeing. As sustainability has not yet been reached, this finding may hinder the long-term sustainability of the reform.

## Discussion

In the following subsections we discuss the findings in relation to the validation of the SADE model (RQ1, see Section 6.1), and the sustainability of the reform (RQ2, see Section 6.2), with concrete implications for researchers and practitioners involved in the implementation and sustainability of DE curricular reforms.

### Modelling the Sustainability of digital education curricular reforms (RQ1)

Given the sparsity and scarcity of the sustainability literature, the SADE model needed to have a strong theoretical foundation. The model therefore built not only upon the literature on sustainability of changes in teachers’ practices, but also i) the evaluation frameworks provided by Guskey (2000), Avry et al. (2022), and the underlying models of ii) acceptance of innovation (Davis, 1985; Venkatesh et al., 2003) and iii) Technological Pedagogical and Content Knowledge (Mishra and Koehler, 2006). The findings support the validity of :

- the measurement model for evaluating sustainability (i.e. the sustainability survey, *χ*^2^(62) = 128, *p* < 0.001, *χ*^2^/*df* < 2, *CFI* = 0.922, *TLI* = 0.902, *RMSEA* = 0.063, *RMSEA* 90*%ci* = [0.047;0.079], *SRMR* = 0.054)
- the second-order Sustainable Adoption of Digital Education (SADE) structural model (*χ*^2^(75) = 144, *p* < 0.001; *χ*^2^/*df* < 2; *CFI* = 0.921; *TLI* = 0.904; *RMSEA* = 0.059; *RMSEA* 90*%ci* = [0.044;0.073]; *SRMR* = 0.054), a first statistically validated model predicting the influence of multiple factors on DE pedagogical content adoption.

The results of the modelling highlight the complexity of DE pedagogical content adoption which requires that a complete ecosystem be implemented and sustained in schools in order to ensure the sustainability of the changes in teachers’ practices. This is because all the items considered load highly on their latent factors (i.e. ease of use, utility, and support). The latent factors, in turn, significantly impact teachers’ sustained adoption of the discipline more than a year after the end of the DE-PD program. Therefore, to promote positive and sustained changes in teachers’ practices, it is essential to provide the required support in schools (standardised effect *β* = 0.183), ensure that teachers are able to easily integrate DE into their practices (standardised effect *β* = 0.275), and that teachers perceive the utility of the reform (standardised effect *β* = 0.275), not only in the first phases of the reform, but also in the long term. To that effect, both the measurement model and the structural model can support researchers and practitioners involved in DE curricular reforms by providing guidelines on how the reform should be implementation, what support is needed, and how it may be evaluated to achieve sustainable changes in teachers’ practices (as done in the present case and detailed in RQ2). The evaluation, however, requires that curricular reforms and professional development programs i) consider more extensive evaluation frameworks that go beyond simply measuring satisfaction or intention and move towards higher levels of Guskey (2000)’s evaluation framework such as the ones provided in the present article, ii) conduct evaluations that persist over time until sustainability is reached, and as such go beyond the implementation phases which most endeavours tend to stop at. Indeed, given the complex dynamics in the field, and the constantly changing demands placed on teachers, if one of these conditions is not met before sustainability is reached, the reform runs the risk of failing in the long term. However, the proportion of variance explained by the model (*R*^2^ = 8.1*%*), suggests that expanding the model to include a wider range of factors would be beneficial to explain teachers’ adoption of DE pedagogical content. Future work could enrich the SADE model by considering other levels of Guskey (2000)’s model, and the factors that contribute to scalability of reforms (Coburn, 2003).

To that effect, the first two levels of Guskey (2000)’s model would benefit from gaining insight into the teachers’ knowledge (Hubers, 2020, e.g. Technological Pedagogical and Content Knowledge, Mishra & Koehler, 2006). Awareness of what teachers have mastery of, and not just their perception of understanding the concepts (as evaluated in the present study), will also help limit the “gap between intended curricula and actually implemented curricula” (Thomas et al., 2009).

From the perspective of adoption (Guskey, 2000’s fourth level), it would be interesting to include the depth of the change in teachers’ practices (Coburn, 2003) and / or content appropriation (Karsenti and Bugmann, 2018; Klingner et al., 2001). This is important because “as a teacher becomes more familiar with the affordances of a particular digital technology, how they integrate the tool will become more specialised and suited to their own teaching and students” (Niederhauser et al., 2018; Drits-Esser et al., 2017). Thus, increased depth is likely to translate into improved student impact, that would feed back into the teachers’ perception of the utility and acceptability (Tricot et al., 2003) of the reform, and increase the likelihood that the reform is sustained (Tikkanen et al., 2020; Vaughn et al., 1998; Klingner et al., 2001; Li, 2017; Jamaludin & Hung, 2016). Additionally, it is unknown to what extent teachers feel they have ownership of the reform (Coburn, 2003), which in this case was imposed by policy makers. Unfortunately, depth and shift in reform ownership are mostly qualitatively evaluated, and to the best of our knowledge, no measurement models exist for these concepts yet (Tan & Hung, 2020).

From the student perspective (Guskey, 2000’s fifth level), it would be interesting to link teachers’ perception of DE curricular reforms with concrete student learning outcomes (Guskey, 2000; Zehetmeier, 2009). PD programs seldom evaluate student outcomes, despite their known impact on the sustainability of the change in teacher practices. This is unfortunate, since “research outputs and dissemination need to be aimed at practitioners (McKenney and Schunn 2018) and able to show a strong connection to learning, to support sustainability and scalability” (Howard et al., 2021). Furthermore, in the context of DE specifically, there are currently numerous difficulties in assessing computational thinking and digital literacy, both for teachers (Roesken-Winter et al., 2015) and students, in large part due to the lack of unified definitions of validated assessments (Godaert et al., 2022; Jamaludin & Hung, 2016).

While each of these factors could each be the subject of their own study, only by investigating all of these dimensions within the same context will we gain insight into the likelihood of achieving a widespread and sustained curricular reform.

Finally, when evaluating the sustainability of educational reforms, one should not forget that teachers are just one of the many stakeholders who play a key role in the endeavour. In fact, the change incurred by a widespread educational reform involves multiple interdependent levels (classroom, school, district, regional, national, and international levels, Kampylis et al., 2013; Hubers, 2020) that should have a common strategic basis and work collectively (Kampylis et al., 2013; Tan & Hung, 2020). The interplay between these different stakeholders should thus be considered when evaluating the sustainability of the reforms (e.g. instructional coaches, school leaders, curriculum designers, trainers, students).

### Sustainability of the digital education curricular reform (RQ2)

The Digital Education curricular reform actively sought to promote sustainability by addressing certain known barriers from the start. This is considered to have contributed to a successful implementation in the first two years of the reform (El-Hamamsy et al., 2021b), and to a *progressive increase of adoption over time*. However, as a successful implementation does not automatically imply that the changes will be sustained in teachers’ practices (Tikkanen et al., 2020; Shirrell & Spillane, 2020), and as sustainable change is “one of the biggest challenges in education” (Hubers, 2020) the findings of the current study help shed light on the sustainability of this curricular reform model. Indeed, investigating teachers’ adoption more than a year after the DE-PD ended further supports the DE curricular reform model which appears efficient from the perspective of sustainability as the reform:

- is relevant and aligned with the needs of the 21st century,
- has political backing and full financial support to provide all teachers in the region *with the necessary PD, as well as adequate pedagogical, and material resources*,
- is led by a multi-institution collaboration between experts and key stakeholders in the region,
- considers school-level specificities by training instructional coaches to provide long-term support to teachers and address school-specific needs (e.g. community of practice, school-specific PD) with the support of school leaders, *factors which are positively perceived by teachers*,
- trains all teachers to introduce the discipline with a DE-PD program that is aligned with the curricular objectives and follows teacher-PD best practices,
- relies on a research-practice partnership to evaluate the outcomes of the curricular reform and PD program to propose recommendations to practitioners for iterative adjustments to ensure the sustainability and scalability of the reform.

However, based on the criteria required to consider that sustainability has been achieved (see Section 3.1), the findings indicate a continuing positive trend, but also that teacher practices have not yet stabilised. Indeed, there is an *increase of adoption over the years*, with nearly 90% of teachers adopting at least one DE activity in the fourth year of the reform (and therefore reaching the expected saturation as one would expect that approximately 10% of teachers will not implement changes, Gersten et al., 2000). However, the cohort of teachers should continue to be monitored in the coming years to ensure that there continues to be an increase in the number of pedagogical activities taught to ensure that all students receive the prescribed amount of DE. Indeed, *teachers consider that the implementation of the DE curriculum still requires effort* (76% consider it costly) and that *they are not yet able to adapt the resources to their pedagogical objectives* (less than 44% consider that they can). A majority would thus *prefer to delegate and have a specialised teacher or instructional coach come and teach the content in their place* (80% in agreement). This is despite the prolonged training and teachers expressing that i) they are capable of teaching the content (self-efficacy, 63% in agreement), ii) have sufficient access to pedagogical and material resources required to teach DE (67% in agreement), and iii) feel supported in their school (77% in agreement). Therefore, the results would suggest that teachers lack the autonomy or motivation to teach the content themselves. However, this may depend on the type of pedagogical activity and the degree to which it is close to teachers’ existing practices (and thus its acceptability, Tricot et al., 2003). In particular, unplugged-type activities that are kinaesthetic and close to the pedagogy employed in primary school appear to be favoured by teachers. It would thus appear important to vary the types of artefacts employed in DE curricular reforms and, in particular, to provide easy entryways to the discipline. This can be achieved by proposing pedagogical activities that employ artefacts that teachers already have a mastery of, i.e., for which they have already achieved the first stage of instrumentation (Trouche, 2005). Doing so could therefore encourage a larger number of teachers to start teaching DE through activities that are closer to their practice. However, there are two possible outcomes if teachers continue to teach DE content:

1. Either teachers continue to adopt activities that do not require altering classroom norms or routines (Vaughn et al., 1998; Coburn, 2003; Sindelar et al., 2006). In that case, the proposed DE pedagogical content should provide the means of covering all the curricular objectives without requiring digital artefacts in primary school (e.g. through unplugged activities).
2. Or the teachers progressively adopt other types of activities (which employ other artefacts) as they become more familiar with DE concepts. Indeed, El-Hamamsy et al. (2021a) found that teachers started adopting unplugged robotics content more frequently a year after their CS-PD (while CS-unplugged activities were already highly adopted the year of the CS-PD). This appears to indicate that more time is required for teachers to appropriate certain types of activities. Please note that this is dependent on teachers having PD-time to learn how to use the instruments (first stage of instrumentation), before learning how to teach with them (second stage of instrumentation, Trouche 2005).

In either case, it is essential to monitor the types of activities that teachers adopt over time and to ensure that students are reaching the DE learning objectives. Given the findings, it may also be relevant to reconsider whether a mandatory DE-PD for all generalist primary school teachers would benefit from being replaced by a specialised-PD for a subset of teachers who would teach DE to all students. This would ensure sufficient DE instruction for all students, until a new generation of teachers who have had DE in their formal education begin teaching DE themselves. Unfortunately, such an approach would not resolve the *lack of in-class-time* to teach DE expressed by teachers. Indeed, in the present context, there is no hour dedicated to teaching DE in the primary school schedule. Teachers are therefore expected to introduce the content transversally, a strategy that is adopted by many countries (European Education and Culture Executive Agency and Eurydice, 2019), although the findings indicate that such a strategy may not promote the sustainability of DE curricular reforms, in addition to a recent study finding that an integrated approach is less likely to promote student learning (Suessenbach et al., 2022). Unfortunately, despite the considerable resources invested in such reforms, the lack of class time represents a significant political barrier (Johnson, 2006) that needs to be addressed.

Finally, teachers are *uncertain that their students are benefiting from the reform* as currently there is no evidence for teachers that the reform leads to positive student outcomes. This is despite multiple studies having shown the importance of teachers seeing that their students are benefiting so that they value the innovation and sustain the change in their practice (Klingner et al., 2001; Howard et al., 2021). However, teachers were not trained to evaluate their students’ competencies in the present context. This appears conjointly with a more global issue: the difficulty assessing competencies in these fields and, in particular, Digital Literacy (related to ICT) and Computational Thinking (related to Computer Science), and thus identifying the utility of teaching the associated pedagogical content. Both Digital Literacy and Computational Thinking suffer from a lack of consensus on what should be assessed and how, with only a limited number of valid and reliable instruments (Roesken-Winter et al., 2015; Godaert et al., 2022; Jamaludin and Hung, 2016). It is not surprising to find that teachers generally struggle to assess their students in these domains even when they are provided the instruments to do so (e.g. as done by Chevalier et al., 2022). Therefore, we believe that i) the difficulty of assessing these competencies, in addition to ii) the lack of training to learn to assess these competencies, and / or iii) access to assessment guidelines, contribute to teachers lacking evidence of student learning, and ultimately being unsure that the students are progressing. PD programs should thus both look to equip teachers with means of assessing their students and provide evidence of the benefits of the specific activities at the student level. For instance, El-Hamamsy et al. (2022b) found that giving teachers feedback on student learning (through figures, descriptive, and inferential statistics) after conducting a CS activity in their classroom helped teachers perceive certain benefits of these activities. However, one should not forget that the impact of DE extends beyond test scores and learning gains (Gu et al., 2019, p. 1119) and that other factors should be accounted for (e.g. developing transversal skills, improving perception, reducing gender biases, and improving disciplinary learning).

### Limitations

As in all studies, the present article is not exempt from limitations. Although we have already expanded on some of them earlier to provide guidelines for researchers and practitioners in these contexts, we provide a synthesis of the limitations here, both overall and per RQ.

Firstly, as in all sustainability studies, such results should be considered context specific (Gersten et al., 2000; Harris and Jones, 2018; Kampylis et al., 2013; Niederhauser et al., 2018). This means the results should be interpreted:

i) within the context of a DE curricular reform that sought to promote sustainability from the start. However, in this case the reform was initiated by policy makers, one must also consider that such an approach “may inspire adoption by teachers or, as Larke demonstrates, they may inspire avoidance” (Gu et al., 2019, p.1119).
ii) with a single cohort (which limits the size of the model that can be tested, and the minimum effect size that can be detected) and at a specific point in time, i.e., in the fourth year of the reform, which is to say more than a year after the two-year DE-PD had ended, with the COVID-19 pandemic in between likely having impacted teachers’ priorities and perception of DE.
iv) within a specific educational system and culture, since “there is substantial diversity between school education systems (Snyder, 2012), [which] can create obstacles when trying to understand progress made in one country and potentially replicate it in another (Hubwieser, 2013)” (Moller & Crick, 2018, p.429).

Secondly, and more specifically with respect to RQ1 and the SADE model, to further validate the model itself, It thus appears relevant to replicate the study over time (e.g. with the same cohort to see how teachers’ needs evolve until sustainability is reached), with larger samples (e.g. in the region as the PD is rolled out to other schools), and in other contexts (e.g. educational systems or countries to determine the generalisability of the findings). The model may also be expanded to include a greater number of factors that are known to influence sustainability of the reform. The survey may also be expanded to include a larger number of items per concept queried, all the while remaining attentive to the fact that the longer the survey, the less likely respondents are to finish answering.

Finally, with respect to RQ2 and the sustainability of the reform itself, as sustainability does not appear to have been reached, such investigations should persist over time to identify whether and to what extent teachers needs evolve over time. The investigations should also consider that adoption is complex and may be modelled by more extensive metrics that account not only for whether a teacher adopted or not, but the extent to which they did it by considering indicators of quantity, completion, and frequency (El-Hamamsy et al., 2022a), and shift to having a classroom-level, rather than a teacher-level adoption metric (as generally two teachers share a classroom in the region). With this information, first-order barriers (i.e. time here) should be addressed by policy makers and sustained over time, while second-order barriers (i.e. perception of the utility of the reform, and identifying the benefits at the student level here) should be addressed by the PD program.

## Conclusion

Sustaining changes in teachers’ practices is a considerable challenge in education, from which Digital Education (DE) curricular reforms are not exempt. Unfortunately, the literature on sustainability is sparse and context-specific, with few studies quantitatively investigating long-term sustainability. Given the increasing number of endeavours introducing novel DE curricula worldwide, it is paramount to investigate the dynamics involved between the various factors at play and understand which significantly impact the sustained adoption of DE pedagogical content. Therefore, the present study investigated the sustainability of a widespread primary school DE curricular reform that actively sought to address sustainability barriers from the implementation phase. We modelled how multiple factors impacted teachers’ sustained adoption of DE pedagogical content drawing from frameworks of professional development evaluation, acceptance of innovation and Technological and Pedagogical Content Knowledge (RQ1), and established whether sustainability had been reached drawing from sustainability criteria provided in the literature, which we adapted to the context of a DE curricular reform (RQ2). The investigation was carried out in the fourth year of the reform, i.e., more than a year after the mandatory two-year DE-PD had ended and was based on data collected from 287 teachers at two points in time during the fourth year of the DE curricular reform.

The analysis is carried out with the proposed Sustainable Adoption of Digital Education (SADE) model, which is based on factors identified in the literature that influence the sustainability of changes in teacher practices. The SADE model groups the factors into three dimensions: perceived ease of use, perceived utility vs. costs, and support provided in the schools. Structural Equation Modelling (RQ1) confirmed the key role of the factors and individual metrics evaluated on the adoption of DE pedagogical content at this stage of the reform. The identified factors should thus be of primary concern and “planned for at the early stages of programme development” (Pieters et al., 2019). To establish whether sustainability has been reached (RQ2), we considered multiple indicators established by Hubers (2020) and operationalised for the context of DE curricular reforms. The teachers’ adoption of pedagogical content shows that i) nearly 90% of teachers are teaching some DE adopting, confirming the adequacy of the curricular reform implementation model (El-Hamamsy et al., 2021b), ii) but also indicates that there are still changes in teachers’ practices four years into the program, and that teachers may still increase the amount of DE taught (RQ2). Furthermore, three main barriers appear, which may still hinder the sustainability of the DE curricular reforms despite the PD, resources, and long-term support in schools. Firstly, the introduction of the DE pedagogical content still appears to require effort on the teachers’ part, with teachers preferring to delegate and have someone else teach it in their place. Secondly, teachers continue to express a lack of time to teach DE in class, despite the fact that DE is in the mandatory curriculum. Finally, the teachers do not have evidence that the students are benefiting from the DE instruction. Combined, these elements may threaten the long-term sustainability of the DE reform as teachers may decide that DE is not a priority worth investing time in, as opposed to other disciplines.

Although the results should be considered context-specific (Gersten et al., 2000; Harris and Jones, 2018; Kampylis et al., 2013; Niederhauser et al., 2018), there are global takeaways that emerge:

- Actively seeking to overcome sustainability barriers from the beginning of curricular reform endeavours is key to successful reform implementation. Indeed, the approach taken to devise the DE curricular reform and pedagogical content was successful in the present case in getting teachers to implement changes in their practices, including in the two years following the end of the DE-PD.
- Multiple sustainability criteria, such as those provided by the SADE model (e.g. related to adoption, perceived utility vs. costs, ease of use, support) and the models from which it draws inspiration, must be monitored until the teachers’ practices have stabilised and positive student outcomes are attained in order to identify teachers’ needs and provide the required support to achieve sustainability of the reform. This implies having i) infrastructures that permit researchers to conduct longitudinal studies with the considered cohort of teachers (e.g. through research-practice partnerships), and ii) having actors (e.g. instructional coaches) that are present in schools to dynamically sustain the reform over time, even when PD-providers are no longer in the picture.
- Despite insufficient time being a known barrier, many countries expect generalist primary school teachers to teach DE content, without allocating time to it specifically. Unfortunately, adding a new discipline without freeing up time elsewhere is unlikely to lead to sustainable changes, especially when the discipline i) requires introducing new technical pedagogical content knowledge and resources, which differ significantly from existing teacher-practices, and ii) is not assessed. This paradigm must evolve in order to promote the sustainability of DE curricular reforms.
- Evidence-based results of student learning must be provided to establish the effectiveness of the specific program from the students’ perspective. Considering that “the literature on school improvement is littered with sombre reports of how ICT-mediated innovations have failed to create impact on teaching and learning” (Toh, 2016, p. 145; Gu et al., 2019, p.1118), and that teachers are not convinced that their students are benefiting, this relies on addressing a major gap in the field of DE: i) developing reliable and validated assessments that lack in the subfields (Computer Science and computational thinking, and digital literacy), and ii) training teachers to assess students with respect to these competencies (Vivian & Falkner, 2018; Redmond et al., 2021), which certain studies have found that teachers presently struggle with (Chevalier et al., 2022).

We believe that these elements require consideration by both researchers and practitioners involved in Digital Education in a co-constructive approach to achieve long-term sustainability of such endeavours.

## Data Availability

The data is publicly available on Zenodo (El-Hamamsy et al., 2022c), 10.5281/zenodo.7406815.

## Footnotes

1 Scalability of educational endeavours here refers to spread as defined by Coburn (2003) in her framework for rethinking scale. Other dimensions of scale are depth of the change, sustainability and shift in reform ownership.

2 The up-to-date DE curriculum is available at https://www.plandetudes.ch/web/guest/education-numeriquehttps://www.plandetudes.ch/web/guest/education-numerique

3 The 2021-2022 version of the DE pedagogical content is available at https://www.vd.ch/fileadmin/user_upload/accueil/Communique_presse/decodage.pdf

4 While Kruskal Wallis is not originally intended for repeated measurements, this test was employed because: (i) Friedman’s test could not be used due to having incomplete data and being unable to assign the adoption to specific individuals, (ii) Wilcoxon’s signed rank test can only compare two groups, (iii) ANOVA requires that the residuals be distributed normally which is not the case here.
